# Novel Lung Cell-Penetrating Peptide Targets Alveolar Epithelial Type II Cells, Basal Cells, and Ionocytes

**DOI:** 10.3390/pharmaceutics17070824

**Published:** 2025-06-25

**Authors:** Jin Wen, Gajalakshmi Singuru, Jeffrey Stiltner, Sanjay Mishra, Kyle S. Feldman, Kayla McCandless, Raymond Yurko, Kazi Islam, Ray Frizzell, Hisato Yagi, Jonathan M. Brown, Maliha Zahid

**Affiliations:** 1 Department of Cardiovascular Medicine, Mayo Clinic, Rochester, MN 55905, USA; wen.jin@mayo.edu (J.W.); singuru.gajalakshmi@mayo.edu (G.S.); 2 Department of Developmental Biology, University of Pittsburgh School of Medicine, Pittsburgh, PA 15213, USA; 3 Department of Pediatrics, University of Pittsburgh School of Medicine, Pittsburgh, PA 15213, USA; skm25@pitt.edu (S.M.);; 4 Clinical Virology Laboratory, Yale New Haven Hospital, New Haven, CT 06510, USA; 5 Peptide and Peptoid Synthesis Facility, University of Pittsburgh, Pittsburgh, PA 15260, USA; 6 Consultant, MPEG LA, L.L.C., Denver, CO 80206, USA

**Keywords:** cell-penetrating peptides, alveolar type II cells, basal cells, ionocytes, siRNA

## Abstract

**Background:** Cell-penetrating peptides cross cell membrane barriers while carrying cargoes in a functional form. Our work identified two novel lung-targeting peptides, S7A and R11A. Here, we present studies on biodistribution, the cell types targeted, and an in vitro proof of application. **Methods:** Studies were performed in human bronchial epithelial cells (HBECs) with and without various endocytic inhibitors, and coincubation with fluorescently labeled transferrin or endocytic markers. Cyclic R11A (cR11A) was conjugated to siRNA duplexes and anti-viral activity against SARS-CoV-2 was tested. Biodistribution studies were performed by injecting wild-type mice with fluorescently labeled peptides, and various circulation times were allowed for, as well as cross-staining of lung sections or isolated single cells with various cellular markers, followed by fluorescence-activated cell sorting or confocal microscopy. **Results:** cR11A showed peak uptake in 15 min, with the highest uptake in airway epithelial type II (ATII) cells, followed by p63+ basal cells and ionocytes. Cyclization increased transduction efficiencies ~100-fold. Endocytosis studies showed a decrease in peptide uptake by pre-treatment with Pitstop2 but not Amiloride or Nystatin. Endocytic marker Lamp1 showed colocalization at the earliest time point, with the escape of the peptide from endocytic vesicles later. cR11A conjugated to ant-spike and anti-envelop proteins showed anti-viral effects with an EC_90_ of 0.6 μM and 1.0 µM, respectively. **Conclusions:** We have identified a novel peptide, cR11A, that targets ATII, basal cells, and ionocytes, the cyclization of which increased transduction efficiency in vitro and in vivo. The uptake mechanism appears to be via clathrin-mediated endocytosis with escape from endocytic vesicles. cR11A can act as a vector to deliver anti-viral siRNA to epithelial cells.

## 1. Introduction

Cell-penetrating peptides (CPPs) are 5–30 amino acid long peptides, capable of breaching cell membrane barriers without a change in cell viability while carrying cargoes much larger than themselves in an intact, fully functional form [1,2]. The first CPP identified was from the HIV coat protein, a trans-activator of transcription (Tat) [3,4]. The cell-penetrating abilities of Tat, an 86-amino acid protein, were mapped to a short 11 amino acid, cationic, arginine- and lysine-rich domain. Subsequent studies showed that Tat could be fused to a much larger marker protein, beta-galactosidase, and, upon systemic administration in wild-type mice, resulted in robust transduction of multiple tissues, even crossing the blood–brain barrier, with diffuse uptake in neuronal tissue. However, such ubiquitous transduction was an obstacle to the development of these non-tissue specific CPPs as vectors for clinical use. This drawback was circumvented by the use of phage display [5]. Using this technique [6], investigators have identified peptides targeting tumors [7], pancreatic islet cells [8,9], adipose tissue [10], synovial tissue [11], and cardiomyocytes [12], to name a few.

Our prior work utilizing an in vitro and in vivo phage display combinatorial methodology [6] led to the identification of a unique, non-naturally occurring, mildly basic, 12-amino-acid-long peptide, which we termed cardiac-targeting peptide (CTP-APWHLSSQYSRT) [12] due to its ability to robustly transduce cardiomyocytes in as little as 15 min, the earliest time point tested, after a retro-orbital intravenous injection [13]. This transduction ability was cardiomyocyte-specific and not species-limited [13,14,15], with our findings confirmed by five independent laboratories from across the world [14,15,16,17,18,19]. As part of a larger set of studies intended to elucidate the mechanism of transduction of this peptide, we performed an alanine scan in which each of the 12 amino acids was sequentially replaced by alanine, which led to the serendipitous identification of two robust, lung-targeting peptides, S7A (APWHLSAQYSRT) and R11A (APWHLSSQYSAT). In the current body of work, we detail the work leading to the identification of these peptides, compare linear and cyclized versions, and show that our lead lung-targeting peptide, cyclic R11A (cR11A), is taken up by lung tissue in as little as 15 min after intravenous injection, and specifically targets alveolar epithelial type II (ATII) cells, the resident progenitor cells critical for post-injury lung regeneration, along with basal cells and ionocytes. We also present data on the mechanism of cR11A’s uptake. Furthermore, as proof of concept, we show that, using a linker with a disulfide bond, oligonucleotides can be covalently conjugated to the N-terminus of this peptide. Lastly, we provide an application of cR11A as a vector for the delivery of siRNA targeting SARS-CoV-2 spike S and envelope E proteins in an air–liquid interface model of viral infection using human bronchial epithelial cells.

## 2. Materials and Methods

**Peptide Synthesis**: Linear peptides were synthesized on a Liberty CEM microwave synthesizer using Fluorenylmethyloxycarbonyl chemistry. The N-terminal amino group of the peptides was conjugated with Cy5.5-NHS (Lumiprobe Corporation, Cockeysville, MD, USA; Cat # 27020). Cyclic Cy5.5-labeled peptides were synthesized as above with lysine added to the N-terminus, followed by head-to-tail cyclization using 3-(diethoxyphosphoryloxy)-1,2,3-benzotriazin-4(3H)-one in Dimethylformamide/Dichloromethane for 5 days at room temperature. The epsilon amino group of lysine was then labeled with Cy5.5-NHS in 0.1 M Triethylammonium bicarbonate/acetonitrile at pH 8.5. The resulting Cy5.5-labeled peptides were purified by preparative C-18 RP-HPLC on a Waters Delta Prep 4000 chromatography system followed by lyophilization. All synthesized peptides underwent MALDI-TOF or LC/Mass Spec (LC-MS) analysis of the purified conjugates for confirmation of the expected mass and identity of the final product. cR11A used in our biodistribution and cell-targeting studies in the lung was >97% pure.

**Fluorescence-activated cell sorting (FACS):** The Rat cardiomyoblast cell line H9C2 (ATCC, Manassas, VA, USA; Cat # CRL-1446) was cultured in high-glucose Dulbecco’s Modified Eagle’s Medium (DMEM; Gibco, Grand Isle, VT, USA) supplemented with 10% fetal bovine serum (FBS) and maintained at 37 °C in a humidified incubator with 5% CO_2_. Human bronchial epithelial cells (HBEC, ATCC, Cat# PCS-300-010) were cultured in Airway Epithelial Cell Basal Medium (ATCC PCS-300-030) supplemented with the ATCC Airway Epithelial Cell Growth kit (ATCC PCS-300-040), according to the supplier’s instructions. For FACS experiments, the cells were seeded in 6-well plates at a density of 1 × 10^5^ cells per well. After 24 h, the cells were incubated with 10 μM of CTP, 1 μM of linear, or 100 nM of cyclic S7A or R11A, and co-incubated with 1 μL/mL of Live/Dead fixable yellow stain (Invitrogen, Carlsbad, CA, USA; Cat # L34968) for 30 min. The cells were then washed with PBS, trypsinized, collected, and fixed with 2% paraformaldehyde (PFA) at room temperature for 10 min. After washing, the cells were resuspended in phosphate-buffered saline (PBS), and analyzed using a BD LSRFortessa™ X-20 Cell Analyzer. Untreated cells, cells treated with live/dead stain alone, and cells treated with 1 μM of a scrambled, random peptide (RAN: TALACYPHTVGQ) were used as controls. All groups were tested in technical quadruplicates, and the experiment was repeated on different days to provide biological triplicates. Gating was performed on cells that were negative for the live/dead stain, thereby excluding dead cells.

**Serum stability studies:** Linear and cyclic R11A were added to fetal bovine serum (FBS) at a final concentration of 10 µg/mL, with 250 µL of this aliquoted into Eppendorf tubes, and placed in a 37 °C/5% CO_2_ incubator for 0, 5, 10, 15, 30, 60, 120, 240 and 360 min. At the end of each incubation time point, the tubes were removed and placed on ice; 25 µL of 1 µM C-peptide as an internal standard was added to the tube and vigorously vortexed for 15 s, followed by the addition of 500 μL of acetonitrile to each tube, vortexing for 30 secs, and centrifugation at maximum speed in a tabletop centrifuge for 5 min at 4 °C. The clear supernatant was transferred to a new tube and analyzed using mass spectrophotometry. A full MS scan was performed from 600.0 to 725.0 m/z (mass/charge) without fragmentations in positive polarity at a resolution of 70,000 (the range option is 17,500–140,000). The maximum injection time was 200 msec. The gas flow rates in arbitrary units were as follows: 48 for sheath, 11 for auxiliary, and 3 for sweep. The spray voltage was 4.00 kV, with a spray current of 5.90µA, a capillary temperature of 320 °C, and an auxiliary gas temperature of 250 °C with the S-lens Radio Frequency (RF) set to 50 (arbitrary units from 0 to 100). The peak area detected for cR11A ranged from 728 to 730 m/+2, with the major ion at 729.36 m/+2. Intensities were quantified as the peak area containing all ion species within the specified m/+2 range. Then, 20 µL of the sample was injected onto an Eclipse Plus C18 column (1.8 um; 2.1 × 50 mm) at 45 °C at a rate of 0.3 mL/min and eluted with a gradient of acetonitrile (in 0.1% formic acid) using water in 0.1% formic acid as the aqueous phase. Column loading was carried out at 5% acetonitrile for 45 s prior to gradient elution. A shallow elution gradient from 5 to 30% acetonitrile occurred over 8.25 min; then, a step to 50% acetonitrile occurred, which was maintained for 1 min. Column re-equilibration to 5% acetonitrile was established for 0.9 min prior to the next injection. cR11A eluted at 6.55 min (23% acetonitrile). All peptides and all times were tested in triplicate.

**Endocytosis studies:** In order to investigate the mechanism of cell entry, human bronchial epithelial cells (HBECs) were serum-starved overnight and subsequently treated with specific endocytic pathway inhibitors. The cells were incubated with Pitstop2 (15 μM to inhibit clathrin-mediated endocytosis), Amiloride (400 μM to inhibit macropinocytosis), or Nystatin (25 μM to inhibit caveolae-dependent endocytosis) for 1 h, followed by the addition of cR11A (1 μM) for 30 min. At the end of the incubation period, the cells were washed, trypsinized, harvested, and fixed with 2% PFA for 10 min at room temperature (RT), followed by a single wash and resuspension in PBS, and they were subjected to FACS analysis. In another set of experiments, HBECs were treated with cR11A at 10 μM for 30 min, followed by the removal of cR11A and replacement with media for varying durations (0, 5, 15, 30, 60, and 120 min). The cells were then fixed with 4% PFA, permeabilized with 1× permeabilization Buffer (eBioscience, San Diego, CA, USA; Cat# 00-8333-56), and blocked with 5% serum in PBS for 1 h at RT. The cells were incubated with anti-LAMP1 antibody (Abcam, Cambridge, UK; Cat# ab24170) overnight at 4 °C, washed, and stained with Alexa-Fluor-488-conjugated secondary antibody at RT for 1 h, mounted with DAPI, imaged in 3D using a Zeiss LSM 980 microscope, and analyzed with Imaris software (version 10.2.0) For the transferrin uptake assay, human Cystic Fibrosis bronchial epithelial cells (CFBE41o-) were grown on coverslips, and was serum starved for one hour at 37˚C. The cells were washed with cold PBS, incubated with Alexa-488-labeled transferrin on ice in a cold room, and incubated for an hour. At the end of incubation, Cy5.5-labeled random, linear or cyclic LTPs were added and transferred to 37˚C, and after each time point, the cells were washed and fixed in 4% PFA. The cells were stained with Hoechst and mounted for confocal imaging.

**Animal studies:** All animal protocols were approved by the University of Pittsburgh and the Mayo Clinic’s Institutional Animal Care and Use Committee. Wild-type, 6-week-old, CD1 (Charles Rivers, Wilmington, MA, USA; Cat #022) mice were weighed and anesthetized with an intraperitoneal injection of a 1:1 cocktail of Ketamine–Xylazine at 1–2 μL/gm body weight, followed by intravenous injection with Cy5.5-labeled peptides that were allowed to circulate for various time points. At the end of the circulation times, the mice were euthanized using inhalational CO_2_, their chest cavity was opened, their right atrium nicked and 3–5 mL of 10% formalin was injected through the left ventricular apex to perfusion-fix the animals. Various organs were harvested, rinsed once in PBS and imaged using an In Vivo Imaging System (IVIS; PerkinElmer Inc., Waltham, MA, USA) with identical instrument settings (stage position, exposure time, binning, F-stop, etc.). The organs were placed in 10% formalin at RT for 4 h, transferred to 15% sucrose at 4 °C overnight, followed by 30% sucrose at 4 °C overnight, before being embedded and cryo-sectioned at 6 μM thickness. The sections were cross-stained with various primary antibodies for ATII cells (ABCA3), basal cells (p63, KRT5), ionocytes (FOXI1), goblet cells (MUC5AC), ATI cells (HOPX), ciliated cells (acetylated tubulin), club cells (Uteroglobin), pulmonary endothelial cells (CD31), and airway smooth muscle cells (α-SMA) overnight at 4 °C. The sections were washed and incubated with Alexa-Fluor-488-conjugated secondary antibody at RT for 1 h. Additionally, kidney sections were stained with Lotus tetragonolobus Lectin-FITC (LTL-FITC) at RT for 1 h. All sections were then washed and mounted with DAPI, and confocal microscopy was performed. All peptides and time points were tested in triplicate (N = 3).

**Isolation of single cells from mouse lung tissue:** After injecting the mice with cR11A as above, lungs were harvested and immediately placed in cold PBS with 2% FBS, cut into small pieces, transferred into a 50 mL tube containing 5 mL of digestion buffer (RPMI 1640 medium with 1× collagenase/hyaluronidase (Stemcell, Vancouver, BC, Canada; Cat# 07912), and DNase I Solution 0.15 mg/mL (Stemcell; Cat# 07900), and incubated at 37 °C for 1 h on a shaking platform at 180 rpm. After incubation, the digested tissue was strained through a 70 µm strainer, rinsed with PBS containing 2% FBS and 1 mM EDTA, filtered again through a 70 µm strainer, and washed with cold PBS containing 2% FBS. The cells were pelleted, washed and treated with 5 mL 1× RBC lysis buffer (BioLegend, San Diego, CA, USA; Cat# 420301) for 5 min, followed by 10 mL PEB buffer (1× PBS + 2 mM EDTA + 1% FBS) to stop the reaction; the cells were pelleted and fixed with 4% PFA for 10 min, washed, and permeabilized with permeabilization buffer (eBioscience, San Diego, CA, USA; Cat# 00-8333-56) at RT for 10 min. After three washes with PBS, the cells were blocked with mouse Fc-receptor blocker (Thermo Fisher, Waltham, MA, USA; Cat# 14-9161-73) and 5% FBS in 0.1% TBST for 1 h at RT; the cells were aliquoted and incubated with the primary antibodies for various cell types (see prior paragraph) at 1:100 dilution at 4 °C overnight. After incubation, the cells were washed and incubated with Alexa-Fluor-488-conjungated secondary antibody at 1:500 dilution and 1 μL/mL of yellow live/dead staining at RT for 1 h, washed, and resuspended in 500 µL PBS for FACS. All FACS analyses were carried out in quadruplicate.

**Design of anti-SARS-CoV-2 siRNA:** An in silico design of SARS-CoV-2-specific siRNA targeting key viral structural proteins, spike S, envelope E, and nuclear N proteins, was performed. We obtained the SARS-CoV-2 complete-genome sequence from GenBank (Accession Number: MN908947.3) [20], and targeted gene-specific siRNA against SARS-CoV-2 structural proteins [21] using siDirect2.0. We selected the optimal 2 siRNAs per target protein, based on thermodynamic stability.

**Conjugation of siRNA to cyclic R11A**: C6-protected siRNA oligomers (IDT technologies) were reduced to their free thiol form using DL-Dithiothreitol in 0.1 M Triethylammonium bicarbonate at pH 8.5 and then reacted with dithio-bis-maleimidoethane in 300 mM NaOAc/acetonitrile at pH 5.2. Purification of the siRNA-DTME intermediate by precipitation with ethanol was followed by reaction with the purified cR11A peptide in 300 mM NaOAc/acetonitrile at pH 5.2. Analytical C-18 RP-HPLC purification of the resulting siRNA-DTME-cR11A conjugate using triethylamine acetate/acetonitrile gradients on a Waters Alliance chromatography system was performed, followed by lyophilization. Confirmation of the expected mass and identity of the final siRNA-cR11A conjugate was carried out by MALDI-TOF analysis on an Applied Biosystems Voyager workstation using a 3-hydroxypicolinic acid matrix in ammonium citrate.

**Anti-viral testing of cyclic R11A-siRNAs:** The antiviral activity of two cR11A-siRNAs was evaluated against SARS-CoV-2 (strain USA_WA1/2020) in a highly differentiated, three-dimensional, in vitro model of normal HBEC inserts (EpiAirwayTM AIR-100, AIR-112) originating from a single donor, # 9981, a 29-year-old, healthy, non-smoking, Caucasian female. Each siRNA-cR11A conjugate was tested for antiviral activity at 3 concentrations (1.8; 0.18; and 0.018 µM) in triplicate. Remdesivir was included as a positive control at 4 concentrations (2, 0.2, 0.02, and 0.002 µM) in triplicate. The cells were cultured at 37 °C for 24 h, and mucin layer was removed by washing with 400 µL of pre-warmed 30 mM HEPES-buffered saline solution. SARS-CoV-2 strain USA-WA1/2020 stock was diluted in AIR-100 mm medium before infection, yielding a multiplicity of infection (MOI) of approximately a 0.0015 cell culture infectious dose, 50% endpoint (CCID50) per cell. Treatment with siRNAs-cR11A (120 µL) was applied to the basolateral and apical side for 24 h prior to infection. For the infection, the virus (120 µL) was applied to the apical side for a 2 h incubation, after which the medium was removed, and replaced with fresh medium. As a virus control, 3 of the wells were treated with cell culture medium only. After 5 days of culture, the virus released into the apical compartment of the culture was collected and plated on Vero 76 cells for virus yield reduction titration. The collected virus was diluted in 10-fold increments and 200 µL of each dilution was transferred into four wells of a 96-well plate to determine 50% viral endpoints. After 5 days of incubation, each well was scored positive for virus if any cytopathic effects were observed as compared with the uninfected control. The virus dose that was able to infect 50% of the cell cultures (CCID50 per 0.1 mL) was calculated by the Reed–Muench method (1938) and expressed as log10 CCID50/mL. Untreated, uninfected cells were used as controls.

**Statistical analysis:** Data are presented as mean ± SEM. Statistical analyses were conducted using GraphPad Prism software (Version 10). For comparisons involving more than two groups, one-way analysis of variance (ANOVA) was performed, followed by Dunnett’s test (for comparisons with a control group) or Tukey’s test (for pairwise comparisons among all groups). For comparisons between two groups, a Student’s *t*-test was used. Statistical significance was defined as *p* < 0.05. Significance levels are indicated as * *p* < 0.05, ** *p* < 0.01, *** *p* < 0.001.

## 3. Results

**Figure 1:**
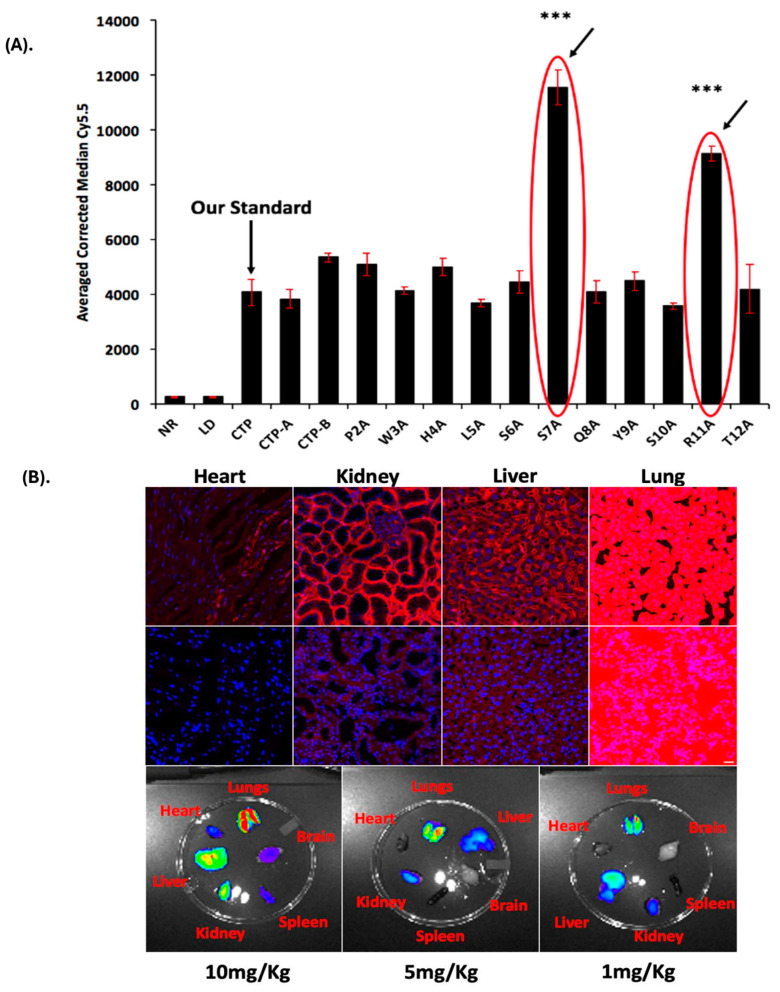
**Identification and biodistribution of lung-targeting peptides.** (**A**). Alanine scan of the original CTP peptide. FACS of H9C2 cells incubated with alanine variants of CTP, and C- and N-terminal six amino acid versions. Cy5.5 intensities were almost 3-fold higher in the S7A and R11A versions. All cell work was carried out in biological triplicates on different days, and technical quadruplicates. Error bars represent standard deviations. *** *p* < 0.001 compared to CTP (our standard) for uptake. (**B**). Lung uptake of S7A and R11A. Wild-type mice were injected intravenously with 10 mg/kg of fluorescently labeled S7A (top row) or R11A (bottom row), euthanized at 15 min, and confocal microscopy of heart, kidney, liver, and lung tissues was performed. The first three panels were imaged using same parameters—the lungs were imaged with significantly shorter exposure times due to fluorescence saturation. Bottom row—mice injected with decreasing doses of R11A (10, 5, and 1 mg/kg), euthanized at 15 min, and ex vivo imaging of multiple organs performed using the IVIS imaging system. There was robust lung uptake of R11A even at the lowest (1 mg/kg) dose. N = 3. Red: peptides; blue: DAPI. Scale bar represents 20 μM.

**Identification and biodistribution of lung-targeting peptides:** Eleven “alanine mutant” versions of CTP were synthesized along with the 6 N-terminus CTP-A (APWHLS) and 6 C-terminus CTP-B (SQYSRT), along with CTP peptide as a standard comparison. H9C2 cells, a rat cardiomyoblast cell line, were incubated with 10 μM of the fluorescent peptides, and FACS was performed (Figure 1A). H9C2 cells were chosen because initial phage display studies that led to the identification of CTP were conducted in this cell line, and we had prior evidence of successful transduction of these cells with CTP, which we used as a reference for comparing the alanine mutants. Two alanine mutant peptides, S7A and R11A, robustly transduced H9C2 cells with 2–3-fold greater uptake than the reference CTP peptide. These were injected intravenously in wild-type CD1 mice at a dose of 10 mg/kg. Counter to our expectations, uptake in the heart was negligible. Instead, there was robust lung uptake of both peptides with some uptake in the liver and kidneys (Figure 1B: S7A top row; R11A middle row). The lung uptake of R11A was greater than that of S7A, with robust lung uptake of R11A even at 1 mg/kg with an increasingly favorable lung-to-liver ratio with decreasing doses (Figure 1B, bottom row; Supplementary Figure S1). Due to this favorable lung-to-liver ratios even at a decreasing peptide dose, we chose to study cR11A further, and those studies form the basis of this manuscript. To confirm the uptake of these peptides by lung epithelial cells, HBECs were incubated with 10 μM of S7A, R11A or RAN for 2, 10, and 30 min. R11A and S7A were taken up at 10 min with uptake increasing at 30 min, with no uptake of RAN by these cells (Supplementary Figure S2).

**Figure 2:**
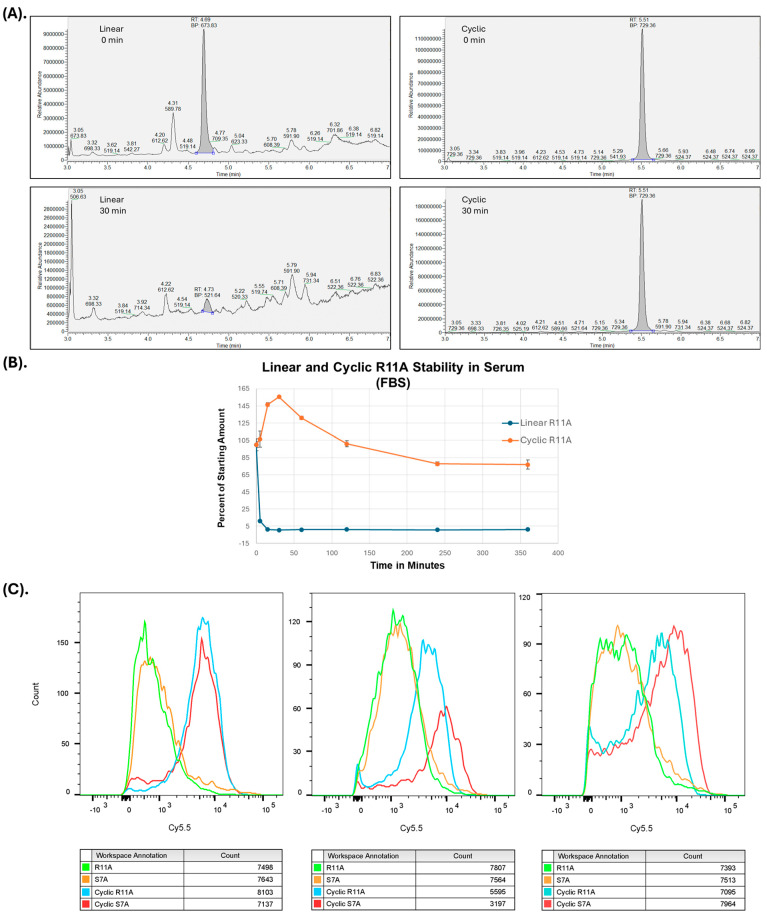
**Cyclization of R11A increases serum stability and transduction.** (**A**). Serum stability studies of linear and cyclic R11A peptides at 0 and 30 min. (**B**). Time course of linear and cyclic R11A stability in serum. (**C**). Cyclization of S7A and R11A increases transduction efficiencies by ~100-fold. Human bronchial epithelial cells were incubated with linear (1 μM) or cyclic peptides (100 nM) and yellow fluorescent live/dead stain, and the intensity of Cy5.5 fluorescence was evaluated on live cells (yellow-fluorescence negative). Cyclic peptides had an ~10-fold increased fluorescence as compared to the linear counterparts at 10-fold lower concentrations. The results shown are for three separate experiments, with 10,000 cells/group evaluated for each experiment.

**Cyclization of S7A and R11A:** There have been multiple reports of cyclic versions of CPPs with higher transduction abilities in vivo compared to their linear counterparts [22,23,24], which the authors felt to be secondary to improved serum stability. To test this hypothesis, we tested the serum stability of linear versus cR11A using mass spectroscopy. Indeed, cR11A had higher serum stability compared to its linear counterpart (Figure 2A, B). The initial multiple peaks seen with linear R11A even at time 0 (Figure 2A, top left panel) show its susceptibility to immediate degradation upon exposure to serum. HBECs were incubated with linear (1 μM) or cyclic peptides (100 nM) for 30 min, and FACS was performed. A lower concentration of cyclic peptides was necessary in order to perform FACS with identical laser settings—the transduction by cyclic peptides was so much more efficient that identical concentrations were off the chart and not able to be compared in a head-to-head fashion. At a 10-fold lower dose, the cyclic peptides still showed an ~10-fold higher cellular uptake as assessed by fluorescence intensities, indicating an ~100-fold increase in transduction efficiencies (Figure 2C).

**Figure 3:**
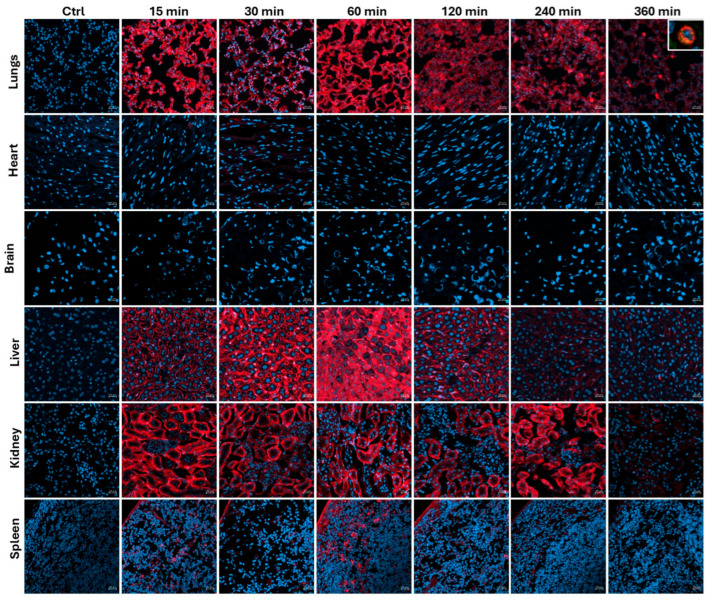
**Biodistribution of cyclic R11A**. Wild-type, 6-week-old, CD1 mice were injected with 1 mg/kg of cR11A-Cy5.5 and allowed to circulate for 15, 30, 60, 120, 240, and 360 min; the mice were euthanized at the end of the circulation period, and confocal micrograph images were taken of the organs, including lungs, heart, brain, liver, kidney, and spleen. The boxed region in the 360 min lung image represents the colocalization of cR11A with FOXI-1, an ionocyte-specific marker. Nuclei are stained with DAPI (blue). Scale bar represents 20 µm (N = 3).

**Biodistribution of cyclic R11A:** Based on these results, we opted to perform a biodistribution study of cR11A. Wild-type, 6-week-old, CD1 mice were injected with 1 mg/kg of cR11A-Cy5.5 and allowed to circulate for 15, 30, 60, 120, 240, and 360 min; these were euthanized at the end of the circulation period using inhalational CO_2_, their right atrium was nicked, the mice were perfused with 10 mL of PBS before being perfusion-fixed with 3 mL of 2% PFA, their organs were harvested, and ex vivo IVIS imaging was performed. The peak uptake by lungs had a bimodal distribution, with uptake at 15 and 60 min. Fluorescence in liver biliary duct cells and renal proximal tubules increased over time, indicating a hepatobiliary and renal mode of excretion, respectively, of either cR11A or its breakdown product(s) that remain fluorescently labeled (Figure 3; Supplementary Figures S3 and S4).

**Figure 4:**
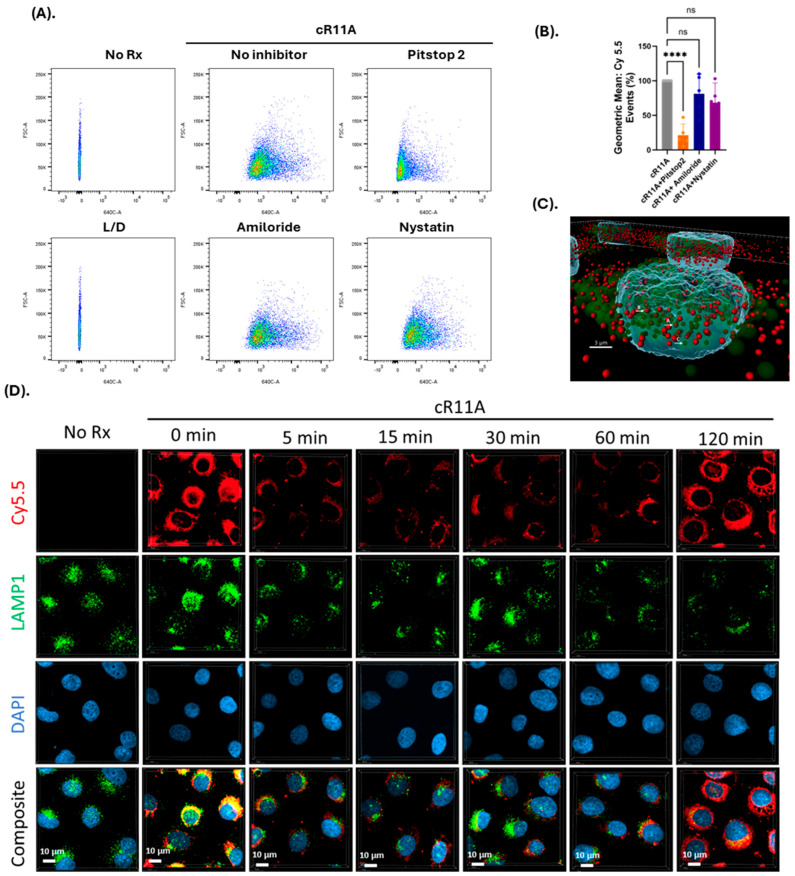
**Mechanism of transduction of cR11A studies.** (**A**). FACS analysis of cR11A uptake by HBECs with or without endocytosis inhibitors. Pitstop2 significantly reduced cR11A uptake, while Amiloride and Nystatin had no effect. (**B**). Quantification confirmed a significant reduction in cR11A uptake with Pitstop2 *(p* < 0.001), whereas Amiloride and Nystatin showed no significant changes (ns) (**** *p* < 0.0001). (**C**). Three-dimensional visualization of colocalization of cR11A and LAMP1. Arrow A: complete co-localization, B: partial co-localization, C: no co-localization. Red: cR11A-Cy5.5, green: LAMP1, blue: DAPI-stained nuclei. Scale bar: 3 µm. (**D**). Confocal microscopy revealed the colocalization of cR11A with lysosomes at 0 min, indicating initial lysosomal uptake. By 120 min, cR11A localized in the cytosol with minimal lysosomal overlap, demonstrating lysosomal escape. Red: cR11A; green: LAMP1; blue: DAPI. Scale bars: 10 μM.

**Figure 5:**
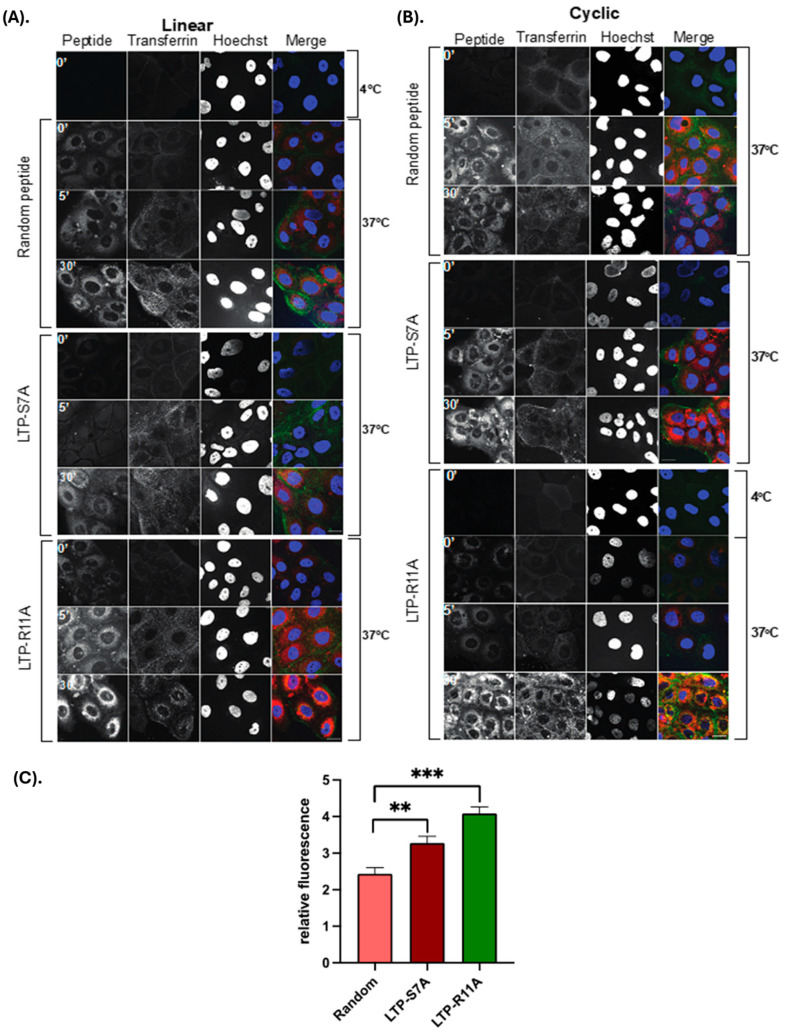
**Cystic Fibrosis bronchial epithelial cells (CFBE41o-) uptake S7A and R11A without colocalization with endocytosed transferrin.** CFBE41o- cells were serum-starved, followed by incubation with transferrin-488, or Cy5.5-labeled linear or cyclic Random, S7A, or R11A, followed by fixation at indicated time points, and confocal microscopy performed. Cells show very little uptake of the R11A and S7A peptide at 4 °C, but increasing uptake at 5 and 30 min, without any co-localization with the green signal of transferrin that was endocytosed into cells. red: peptides; green: transferrin; blue: DAPI. scale bars represent 20 µm. (**A**). Linear peptide colocalization. (**B**). Cyclic peptide colocalization. (**C**). Quantification of relative fluorescence of cyclic peptides. ** *p* < 0.01, *** *p* < 0.001.

**Studies into the mechanism of transduction:** In order to investigate whether the mechanism of uptake involved endocytosis, HBECs were treated with cR11A with or without various endocytosis inhibitors. The uptake of cR11A was significantly reduced by treatment with Pitstop2, but not with Amiloride or Nystatin (Figure 4A,B; Supplementary Figure S5). In order to explore this relationship further, cR11A uptake and cellular localization was tracked relative to lysosomes (labeled with LAMP1). Our experiments revealed high colocalization of cR11A with LAMP1 at the earliest time point (0 min), with reappearance of cR11A in the cytosol at 120 min independent of lysosomes, indicating lysosomal escape of it after cellular uptake (Figure 4C, D). To confirm this last finding, HBECs were serum-starved to stimulate endocytosis, and co-incubated with Alexa-488-transferrin along with S7A, R11A, cR11A, cS7A, or RAN, and confocal microscopy was performed. Our results show that the peptides were internalized and did not show co-localization with the green fluorescence of endocytosed transferrin, indicating that the peptides were indeed escaping endocytic vesicles (Figure 5).

**Figure 6:**
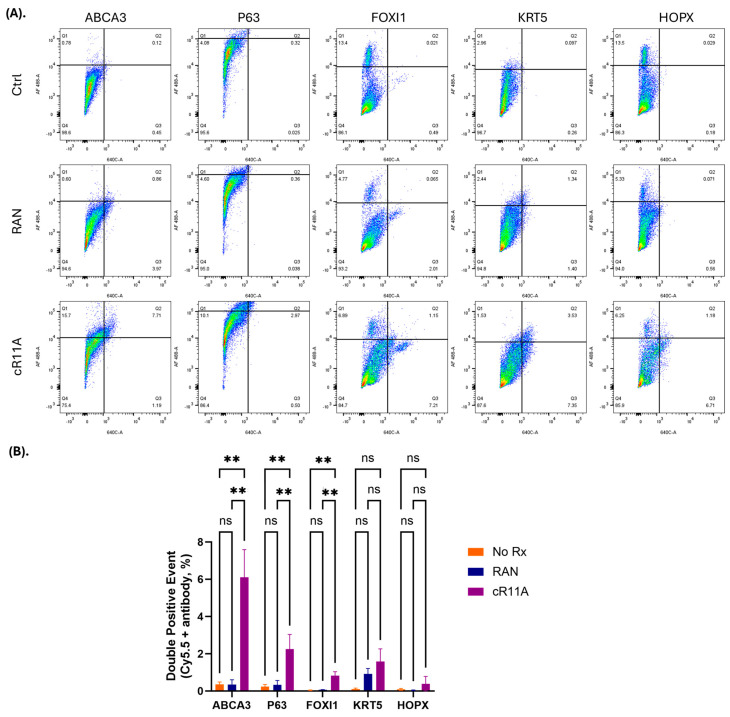
**In vivo lung cell type targeting by cR11A.** (**A**). FACS analysis of lung single cells isolated from wild-type mice injected with cR11A (1 mg/kg), RAN (1 mg/kg), or PBS. Staining with various lung cell markers revealed significantly increased uptake of cR11A in ABCA3^+^ ATII cells, followed by p63+ basal cells, and ionocytes compared to controls. (**B**). Quantification of double-positive events (cR11A-Cy5.5+ and marker+) showed significant uptake in ABCA3^+^ cells, p63^+^ cells and FOXI1^+^ cells in the cR11A-treated group. No significant differences (ns) were observed for KRT5 or HOPX^+^ cells. ** *p* < 0.01.

**Figure 7:**
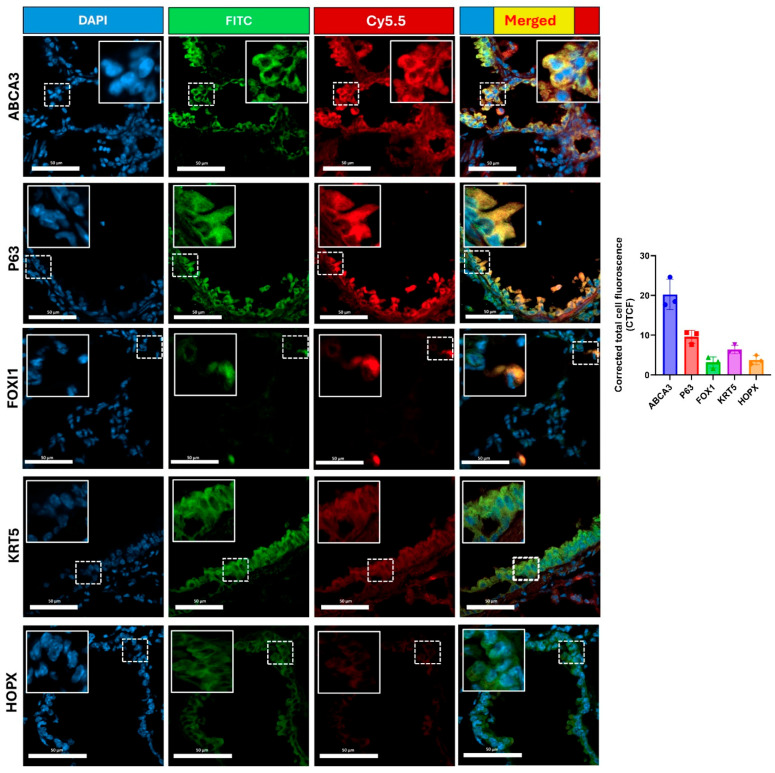
**cR11A targets lung ATII cells, basal cells and ionocytes.** Confocal micrograph of lungs from wild-type mice injected with cR11A-Cy5.5 and euthanized at 15 min. Robust uptake of cR11A (red) is observed in AT-II, basal cells and ionocytes, as shown by co-localization with ABCA3, P63, and FOXI1 (all green). Nuclei are stained with DAPI (blue). Scale bar represents 20 µm (N = 3).

**Lung cell type targeting by cR11A:** To investigate the cell type in the lungs being targeted, wild-type mice were injected with cR11A or RAN at 1 mg/kg, or PBS, and lungs were harvested after 15 min (Supplementary Figure S6) for cryosectioning or single-cell isolation for staining with various cellular markers (Supplementary Table S1). Our single-cell FACS indicated that ATII cells had the greatest uptake of cR11A with 33% of cells staining positive for ABCA3A also positive for Cy5.5-labeled cR11A (calculated by [Q2/Q1 + Q2] × 100), followed by p63+ cells (~23% double-positive) and ionocytes (14% double positive) (Figure 6; Supplementary Figure S7). Interestingly, cR11A was retained by ionocytes as far out as 6 h, the last time point tested (Figure 3, top row insert). Confocal microscopy of these lung sections confirmed our findings of highest colocalization of the cR11A (red) with ABCA3 (green), an ATII cell marker, followed by p63+ (basal cells), and ionocytes (Figure 7; Supplementary Figure S8). Linear R11A (10 mg/kg) showed a lower degree of uptake compared to cR11A (Supplementary Figure S9), similar to our in vitro findings (Figure 2).

**Figure 8:**
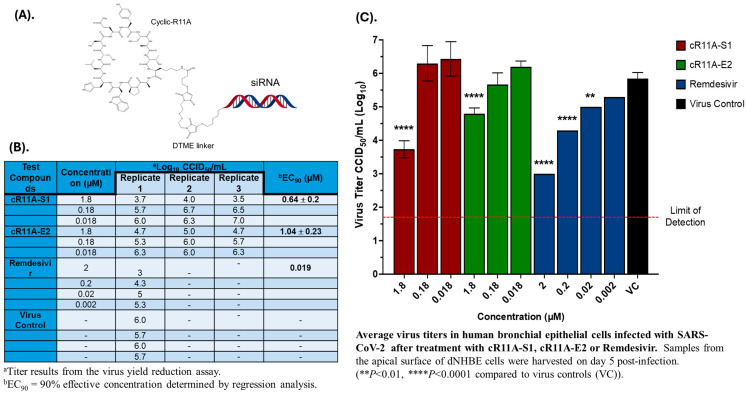
**In vitro antiviral activity of cR11A-siRNA-S1, and cR11A-E2 against SARS-CoV-2 in human bronchial epithelial cell line.** (**A**). Structural representation of the cR11A along with the DTME linker linking to the siRNA represented by double strands. (**B**). Table showing viral titers from human bronchial epithelial cells treated with the cR11A-siRNA conjugates for 24 h prior to infection with SARS-CoV-2 virus and the respective EC90 values, along with the EC90 value for remdesivir. (**C**). SARS-CoV-2 viral titers plotted for cR11A-S1, cR11A-E2, and remdesivir against the virus-only control. All infections and assays were carried out in triplicate.

**cR11A as vector for siRNA delivery:** As proof of concept, we took two optimized siRNA per target proteins of the SARS-CoV-2 virus based on thermodynamic stability (Supplementary Table S2) as possible anti-COVID therapy, and conjugated these to the N-terminus of cR11A via a DTME intermediate that contains an internal disulfide bond (Figure 8A), which has been used to link multiple siRNAs together [25]. Our rationale was that cR11A would internalize the siRNAs, and the disulfide bond would subsequently be cleaved in the reducing intracellular environment, releasing the cargo siRNA to knock down target viral mRNA via the RISC complex, arresting viral replication. The success of the conjugation was confirmed by MALDI-TOF analysis (Supplementary Figure S10). Percent toxicity, as well as percent cytopathic effects of the cR11A-siRNA conjugates in VERO cell lines, was tested by pre-treating with the conjugates for 24 h prior to infection with SARS-CoV-2 virus. Of the conjugates tested, cR11A-S1 and cR11A-E2 appeared to be most promising (Supplementary Table S3) in VERO cell lines. Therefore, the antiviral activity of these two conjugates was evaluated further against SARS-CoV-2 (strain USA_WA1/2020) in an in vitro air–liquid interface of normal human bronchial cells. cR11A-S1 inhibited viral replication with an EC90 value of 0.64 ± 0.2 µM, while cR11A-E2 inhibited virus replication with an EC90 value of 1.04 ± 0.2 µM; for comparison purposes, Remdesivir inhibited viral replication with an EC90 value of 0.019 µM (Figure 8B,C).

## 4. Discussion

In contrast to deep-seated organs like the heart and brain, lungs enjoy a dual route of administration via inhalational or systemic delivery. Although the inhalational route has distinct advantages, like decreased dose requirement, fewer systemic side effects, and the bypassing of the hepatobiliary system, it is not a feasible route in conditions riddled by thick mucus secretions, and a hallmark of pathologies, like cystic fibrosis, asthma, chronic obstructive pulmonary disease, or primary ciliary dyskinesia, that result in the entrapment of drugs. Another factor to consider is that a large fraction of inhaled drug is deposited in the upper airways and may not reach the terminal bronchioles or alveolar epithelial cells. Gene-based therapeutics for lung disorders have not been developed due to the presence of inherent physical barriers like surface mucus, mucociliary clearance, cell-to-cell tight junctions, and the basolateral cell membrane location of viral receptors for many commonly used viral vectors. In addition, viral vectors are associated with either pre-existing immunity or the development of neutralizing antibodies on first exposure, along with potentially lethal side effects like the cytokine release syndrome [26,27]. In contrast, CPPs have not been reported to trigger immune responses [28,29,30,31]. In fact, our data with the parent CTP have shown significant anti-inflammatory effects (unpublished data). Hence, CPPs that specifically target lung tissue would represent viable, alternative vectors that could bypass this bottleneck and lead to the clinical realization of multiple therapeutics.

Our current body of work has identified a unique, 12-amino-acid-long CPP, cR11A, that targets 33% of ATII cells of the lungs after intravenous delivery, as well as p63+ basal cells (22%) and ionocytes (14%) at the dose and time point (15 min) tested, though higher uptake is possible at 60 min. As a reference, ATII cells represent ~15% of the total lung cell population [32]. Interestingly, uptake by ionocytes was retained as late as 6 h, the last time point tested (Figure 3, insert). In vitro uptake of cR11A by human bronchial epithelial cells appears to be mediated by clathrin-mediated endocytosis, with escape from these vesicles at a later time point. We have direct evidence of it by confocal microscopy, as well as indirect evidence in the form of delivered anti-SARS-CoV2 siRNA retaining its anti-viral activity. This would not be possible if there was endosomal entrapment of the cR11A-siRNA conjugate, as the inside of an endosome is still outside of the cytoplasm. This endosomal escape mechanism has been reported for other CPPs, postulating an endosomal budding and collapse mechanism [33,34,35].

We chose to follow detailed biodistribution of cR11A over S7A, as the former showed uptake even at the lowest 1 mg/kg dose, with lung-to-liver ratios increasing with lowering of the dose. The lung uptake of cR11A occurred in as little as 15 min, which is similar to that observed with CTP, where the peak uptake also occurred at 15 min, the earliest time point tested [13]. At later time points, fluorescence in the liver and kidneys is observed, with the pattern suggesting hepatobiliary and renal tubular excretion, respectively. Interestingly, there was a bimodal distribution pattern, with fluorescence in the lungs showing a second peak at 60 min. We compared the cyclized version of both S7A and R11A to their linear counterparts and found that cyclization increased serum stability and uptake by ~100-fold, findings similar to other investigators’ experiences [35,36,37]. cR11A did not cross the blood–brain barrier, with no fluorescence seen in brain tissue on IVIS imaging or confocal microscopy. This is an important observation in light of the recent report of cationic peptides (like Tat or homopolymers of arginine) causing memory loss in mice [38]. The most exciting finding of our study is that cR11A preferentially targeted one-third of all ATII cells in vivo, as well as basal cells and ionocytes, with prolonged retention of cR11A by ionocytes. ATII cells are the resident lung progenitor cells with stem-cell-like properties, and are responsible for the regeneration and replacement of type I alveolar cells in response to myriad different insults [39,40,41], playing a key role in lung regeneration [42]. Additionally, they produce surfactant proteins to reduce lung surface tension and maintain alveolar patency. Recent investigations have identified cellular senescence of AT-II as a critical cause of their stemness failure [43] associated with telomere shortening [44]. Indeed, senescence, apoptosis, and a decreased number of ATII cells play a key role in the pathophysiology of conditions like idiopathic pulmonary fibrosis [43] and chronic obstructive pulmonary disease [45]. Lastly, as proof of concept, cR11A was selected to carry duplex siRNA conjugated to its N-terminus via a DTME linker, targeting various structural proteins of SARS-CoV-2 virus. RNA interference as a therapeutic strategy against SARS-CoV-2 has been demonstrated by others [46,47], utilizing lipid nanoparticles [48,49] and peptide-dendrimers [50]. The advantages of our approach are the use of a lung-specific CPP, and the possibility of systemic delivery bypassing the mucus barrier, with its transduction capabilities further optimized via a cyclization strategy. Our proof-of-concept studies showed anti-viral activity for an siRNA targeting the spike and one targeting the envelope protein.

In the pulmonary space, the development of CPPs has been largely carried out in the context of targeting lung cancers [51,52]. One strategy to target lung cancer takes advantage of the tumor microenvironment by engineering non-tissue specific CPPs and activating them in the tumor microenvironment. This was achieved by neutralizing the polycationic structure of these peptides via linkage to polyanionic peptides via a cleavable linker. These linkers were designed to take advantage of greater metalloproteinase 2 expression in the tumor microenvironment [53], or greater oxidative stress, leading to cleaving of the neutralizing peptide and unmasking of the CPP [54]. In another application, a nine-amino-acid-long cyclic peptide, CARSKNKDC, was reported as targeting multiple layers of pulmonary arteries in a rat model of pulmonary hypertension [55], and was used to deliver micelles containing fasudil, a pulmonary hypertension drug, to the pulmonary endothelium [56]. This uptake was limited to pulmonary endothelial cells and did not target deeper lung tissue. More recently, Soto and colleagues used phage display to identify a CPP targeting HBECs, incorporating it into lipid nanoparticles to deliver mRNA to bronchial epithelial cells in wild-type mice [57]. However, the conjugates were delivered intra-tracheally in wild-type mice, making it difficult to extend the findings to cystic fibrosis lungs with a thicker mucus layer.

Our study has several exciting, seminal findings. To the best of our knowledge, tissue-specific CPPs targeting ATII cells in vivo has not been reported before. This could open up a field of myriad, pathophysiologically driven, disease-modifying therapeutic cargoes being delivered in conditions where underlying ATII cell defects play a leading role. Additionally, the ability of cR11A to target ionocytes after intravenous injection has profound implications for cystic fibrosis due to these cells having a high expression of CFTR protein [58], and their ability to mediate chloride absorption across the airway epithelium [59]. cR11A could be utilized to deliver anti-sense oligonucleotides, like Eluforsen, to correct the splicing mutation in F508del cystic fibrosis [60]. In the CPP field, a multitude of investigators have reported mixing siRNA/miRNA with CPPs to generate nanoparticles by leveraging charge interactions between non-tissue-specific positively charged peptides and negatively charged oligonucleotides. For this work, we developed chemistries to allow for a 1:1 conjugation of CPPs to oligonucleotides through a disulfide-containing linker that is hydrolyzed in the intracellular reducing environment releasing the cargo. In addition to the current work, we have utilized this strategy to deliver miRNA106a using CTP to a human cardiomyocyte cell line in vitro and shown the reversal of hypertrophy with down-regulation of calcium calmodulin kinase IIδ [18].

Our studies have identified several new lines of inquiry, topmost of which is studying the ability of cR11A to deliver siRNA in vivo, as well as its specific mechanism of transduction. Additionally, further investigation of cS7A is needed to identify the specific lung cell types targeted by this peptide. These form the basis of our ongoing studies.

## 5. Conclusions

We have identified a novel cyclic CPP, cR11A, that targets ATII, p63+ cells and ionocytes after intravenous delivery in as little as 15 min. We have successfully conjugated cR11A to oligonucleotides and demonstrated an anti-viral effect in an air–liquid interface bronchial epithelial cell culture.

**Concluding message:** Cell-penetrating peptides cross cell–membrane barriers while carrying cargoes many-fold larger than themselves in a fully functional form. We identified a cyclized peptide that targets lung alveolar epithelial type II cells, basal cells, and ionocytes in vivo, and showed its ability to deliver siRNA ex vivo in an air–liquid interface culture.

## Data Availability

All data relevant to this work is presented in the manuscript and supplemental material.

## Supplementary Materials

The following supporting information can be downloaded at: https://www.mdpi.com/article/10.3390/pharmaceutics17070824/s1, Figure S1: R11A has superior lung targeting abilities than S7A. Wild-type mice injected with 10 mg/kg of Cy5.5 labeled S7A or decreasing doses of R11A (10 mg/kg, 5 mg/kg and 1 mg/kg). Mice were injected with peptides, euthanized at 15 min, and multiple organs harvested for ex-vivo IVIS imaging followed by embedding, cryosectioning, counterstaining with DAPI and confocal microscopy. N = 3 for each dose. Robust uptake of R11A by lung tissue is observed at even the lowest R11A dose of 1 mg/kg with lung to liver ratios improving consistently with lowering of the R11A dose as compared to S7A (lung-to-liver ratio of ~2.5 for S7A (5 mg/kg) compared to 8 for same dose of R11A). Figure S2: Human bronchial epithelial cells transduce robustly with LTPs. Human bronchial epithelial cells were plated on cover slips and treated with 10 μM of linear R11A, S7A or a scrambled random (RAN) peptide for indicated time points at 37 °C, washed 3x with pre-warmed PBS, fixed, counterstained with DAPI and confocal microscopy performed. Both R11A and S7A are robustly internalized by cells by 30 min, and appear to have a cytoplasmic, peri-nuclear localization. Random peptide has very little to no uptake. Figure S3: Biodistribution studies of cR11A in vital organs over time. (A) Representative ex vivo IVIS images of organs from wide type CD1 mice following intravenous injection of cR11A (1 mg/kg) at various time points: 0 min (control), 15 min, 30 min, 60 min, 120 min, 240 min, and 360 min. (B) Organs are arranged in the following order: heart, lungs, brain, liver, spleen, and kidney. (C) Graph showing the average radiant efficiency of heart, lungs, brain, liver, spleen at different time points. (D) Statistic analysis of the average radiant efficiency of lungs over time. * *p* < 0.05, ** *p* < 0.01, **** indicates *p* < 0.0001, compared to the 1-h time point (N = 3). Figure S4: Colocalization of cR11A-Cy5.5 and renal proximal tubules, visualized using Lotus Tetragonolobus lectin (LTL) staining. Kidneys were harvested from mice following an intravenous injection of cR11A (1 mg/kg) at various time points post-injection: 0 min (control), 15 min, 30 min, 60 min, 120 min, 240 min, and 360 min. LTL-FITC staining specifically marks renal proximal tubules, demonstrating the colocalization of cR11A with renal proximal tubules, but not much with glomerulus or renal distal tubules. Cy5.5 (red) represents cR11A, LTL-FITC (green) marks renal proximal tubules, and DAPI (blue) stains nuclei. The scale bar represents 20 µm. N = 3. Figure S5: Transduction mechanism study of cR11A with endocytosis inhibitors. HBE cells were starved and treated with endocytosis markers, Pitstop2, Amiloride, and Nystatin. Subsequently, cells were incubated with cR11A (1 μM), with or without the inhibitors, and subjected to L/D staining. FACS data (640C-A channel) indicated the uptake of cR11A, and L/D staining assessed cell viability. Compared to group treated with cR11A alone (no inhibitor), Pitstop2 significantly reduced cR11A uptake, whereas Amiloride and Nystatin showed no notable inhibition. Results of each biological replicate (N = 5) presented individually. Figure S6: Peak cR11A uptake occurs at 15 min. Wild-type mice were injected intravenously with R11A-Cy5.5 at 1 mg/kg and euthanized, lungs harvested and IVIS imaging performed. There is robust lung uptake of the peptide at 15 min. N = 3. Figure S7: Characterization of lung single cells isolated from cR11A-treated mice. Single cells from lungs of mice injected with cR11A (1 mg/kg) were isolated and stained with markers for various cell types. A) Representative FACS panels display the expression of cell-specific markers, including club cells (Uteroglobin), ciliated cells (acetylated tubulin), goblet cells (MUC5A), pulmonary endothelial cells (CD31), and airway smooth muscle cells (α-SMA). B) Statistical analysis revealed no significant differences in uptake of random peptide from cR11A. Data are presented as mean ± SEM, N = 4. Figure S8: Confocal micrograph of lungs from wild-type mice injected with cR11A-Cy5.5 and euthanized at 15 min. Minimal uptake of cR11A (red) is observed in club, ciliated, goblet, endothelial, and smooth muscle cells, as shown by lack of significant co-localization with acetylated tubulin, MUC5AC, CD31, and alpha SMA (green). Nuclei are stained with DAPI (blue). Scale bar represents 20 µm. N = 3. Figure S9: Confocal micrograph of lungs from wild-type mice injected with linear R11A (10 mg/kg) and euthanized at 15 min. There is uptake of linear R11A (red) in same lung cell types as cR11A but in much lesser amounts, observed in AT2, p63+ cells (green), with no uptake observed in ionocytes, AT-1, club, ciliated, goblet, endothelial, and smooth muscle cells. Nuclei are stained with DAPI (blue). Scale bar represents 20 µm. N = 3. Figure S10: MALDI-TOF analysis of cyclic R11A-siRNA-S1 showing the size and peaks of the conjugate. Sense strand-DTME-cR11A is seen as a single late appearing peak with anti-sense strand breaking off from the conjugate due to ionization processing for MALDI. Excess peptide is seen as the earliest peak. Table S1: List of antibodies for immunofluorescence staining. Table S2: List of duplex siRNA and their targets tested in our study. Table S3: Results of VERO Cells incubated with cyclic R11A-siRNA conjugates followed by infection with SARS-CoV-2 virus. Ref. [61] are cited in the Supplementary Materials.
