# Supplementary material for: Novel Lung Cell-Penetrating Peptide Targets Alveolar Epithelial Type II Cells, Basal Cells, and Ionocytes
Source: Pharmaceutics. 2025 Jun 25;17(7):824. doi: 10.3390/pharmaceutics17070824 (PMC12298391; doi:10.3390/pharmaceutics17070824)
Supplement: Supplementary file 1 [file pharmaceutics-17-00824-s001.zip › pharmaceutics-3712261-supplementary.pdf]

Novel Lung Cell-Penetrating Peptide Targets Alveolar Epithelial Type II Cells, Basal Cells, and Ionocytes

Jin Wen [1,†], Gajalakshmi Singuru [1,†], Jeffrey Stiltner [2], Sanjay Mishra [3], Kyle S. Feldman [4], Kayla McCandless [2], Raymond Yurko [5], Kazi Islam [5], Ray Frizzell [3], Hisato Yagi [2], Jonathan M. Brown [6] and Maliha Zahid [1,*]

[1] Department of Cardiovascular Medicine, Mayo Clinic, Rochester, MN 55905, USA; wen.jin@mayo.edu (J.W.); singuru.gajalakshmi@mayo.edu (G.S.)
[2] Department of Developmental Biology, University of Pittsburgh School of Medicine, Pittsburgh, PA 15213, USA; jstiltnerjr@gmail.com (J.S.); mccandlessk97@gmail.com (K.M.); hisato@pitt.edu (H.Y.)
[3] Department of Pediatrics, University of Pittsburgh School of Medicine, Pittsburgh, PA 15213, USA; skm25@pitt.edu (S.M.); frizzell@pitt.edu (R.F.)
[4] Clinical Virology Laboratory, Yale New Haven Hospital, New Haven, CT 06510, USA; kyle.feldman@ynhh.org
[5] Peptide and Peptoid Synthesis Facility, University of Pittsburgh, Pittsburgh, PA 15260, USA; yurko@pitt.edu (R.Y.); kazi@pitt.edu (K.I.)
[6] Consultant, MPEG LA, L.L.C., Denver, CO 80206, USA; jbrown@mpegla.com
* Correspondence: zahid.maliha@mayo.edu; Tel.: +1-507-255-4622; Fax: +1-507-255-7070
† These authors contributed equally to this work.

Supplemental Results

Supplemental Figure S1.

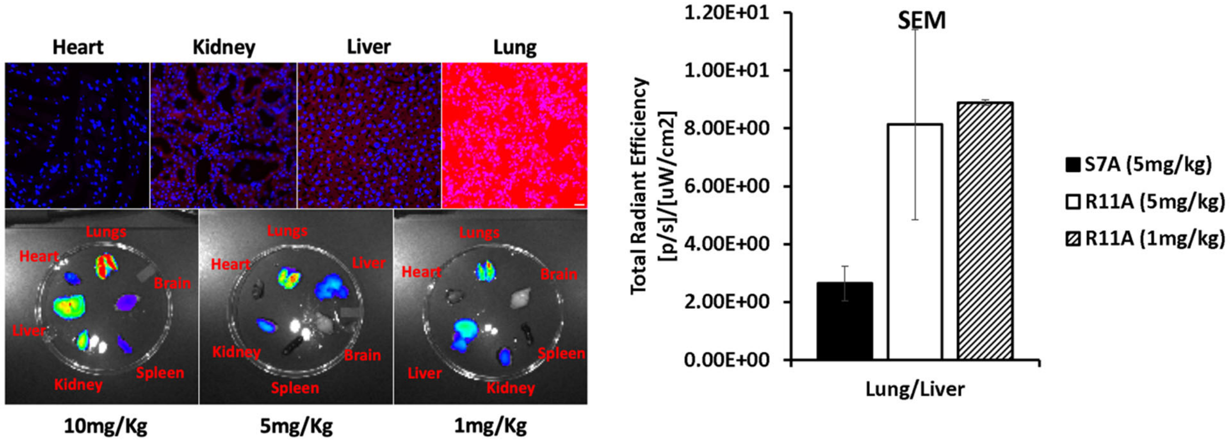

Supplemental Figure S1: R11A has superior lung targeting abilities than S7A. Wild-type mice injected with 10mg/Kg of Cy5.5 labeled S7A or decreasing doses of R11A (10mg/Kg, 5mg/Kg and 1mg/Kg). Mice were injected with peptides, euthanized at 15 mins, and multiple organs harvested for ex-vivo IVIS imaging followed by embedding, cryosectioning, counterstaining with DAPI and confocal microscopy. N=3 for each dose. Robust uptake of R11A by lung tissue is observed at even the lowest R11A dose of 1mg/Kg with lung to liver ratios improving consistently with lowering of the R11A dose as compared to S7A (lung-to-liver ratio of ~2.5 for S7A (5mg/Kg) compared to 8 for same dose of R11A).

Supplemental Figure S2.

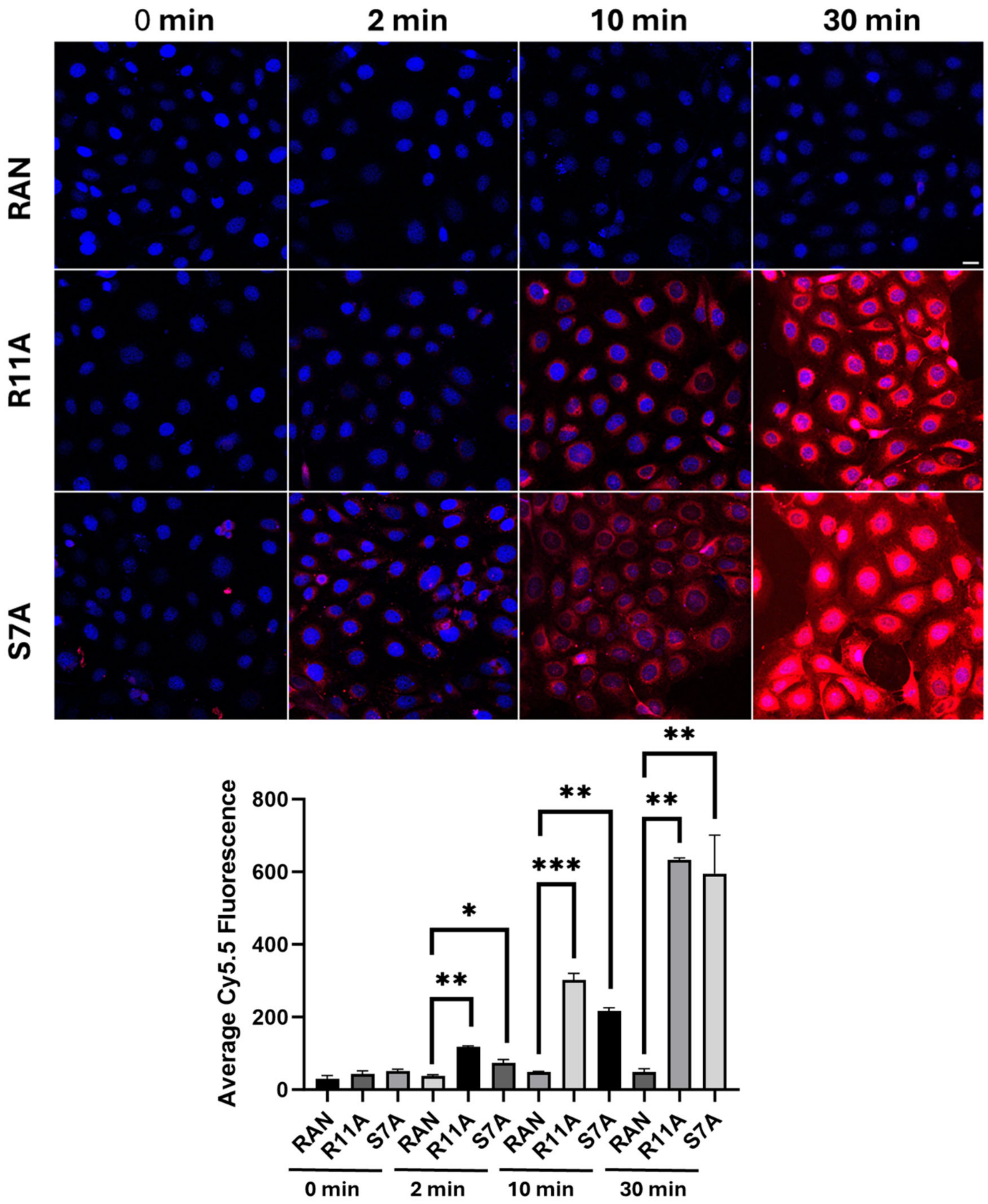

Supplemental Figure S2: Human bronchial epithelial cells transduce robustly with LTPs. Human bronchial epithelial cells were plated on cover slips and treated with 10µM of linear R11A, S7A or a scrambled random (RAN) peptide for indicated time points at 37°C, washed 3x with pre-warmed PBS, fixed, counterstained with DAPI and confocal microscopy performed. Both R11A and S7A are robustly internalized by cells by 30 mins, and appear to have a cytoplasmic, peri-nuclear localization. Random peptide has very little to no uptake.

Supplemental Figure S3.

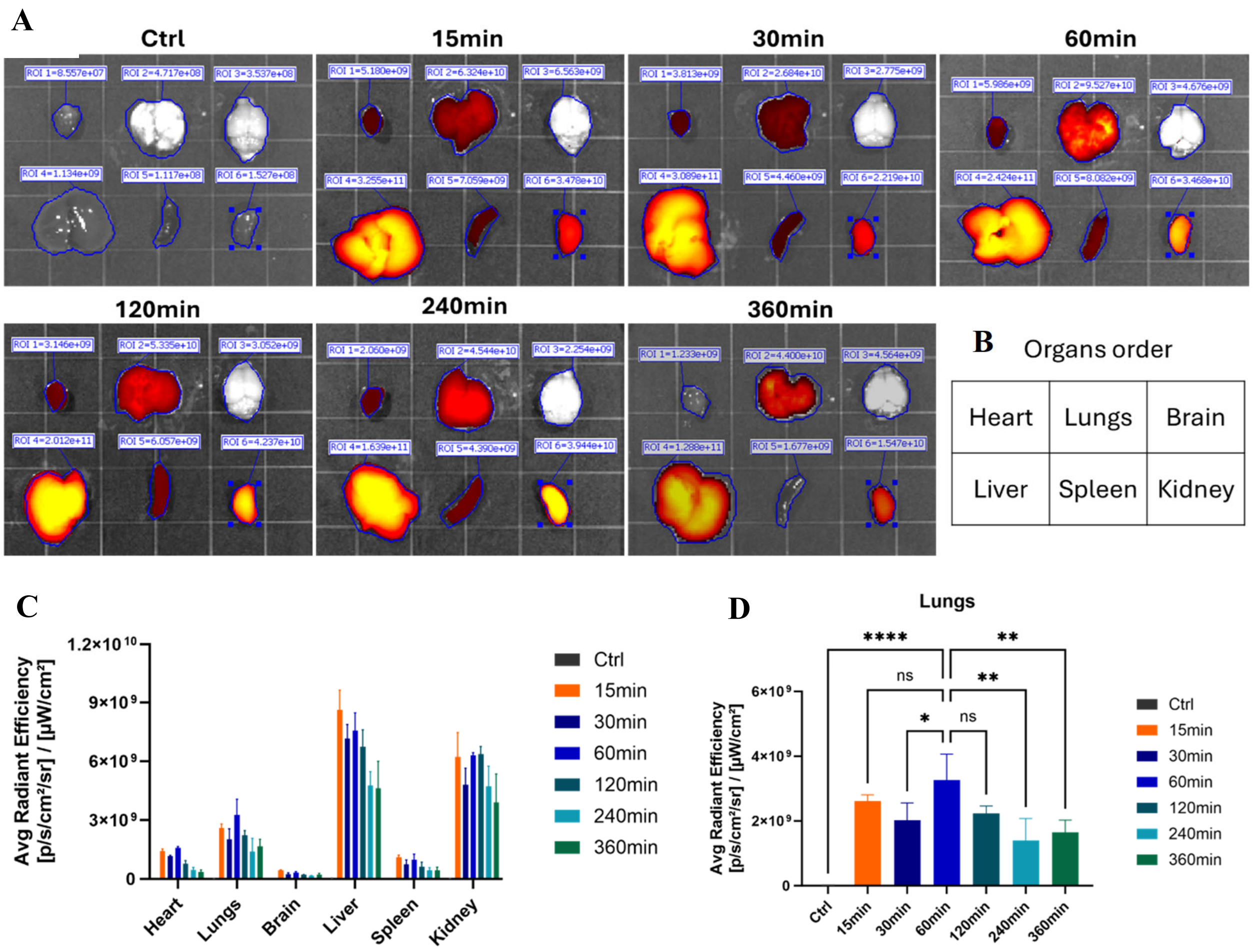

Supplemental Figure S3: Biodistribution studies of cR11A in vital organs over time. (A) Representative ex vivo IVIS images of organs from wide type CD1 mice following intravenous injection of cR11A (1mg/kg) at various time points: 0min (control), 15min, 30min, 60min, 120min, 240min, and 360min. (B) Organs are arranged in the following order: heart, lungs, brain, liver, spleen, and kidney. (C) Graph showing the average radiant efficiency of heart, lungs, brain, liver, spleen at different time points. (D) Statistic analysis of the average radiant efficiency of lungs over time. *$p<0.05$, **$p<0.01$, ****indicates $p<0.0001$, compared to the 1-hour time point (N=3).

Supplemental Figure S4.

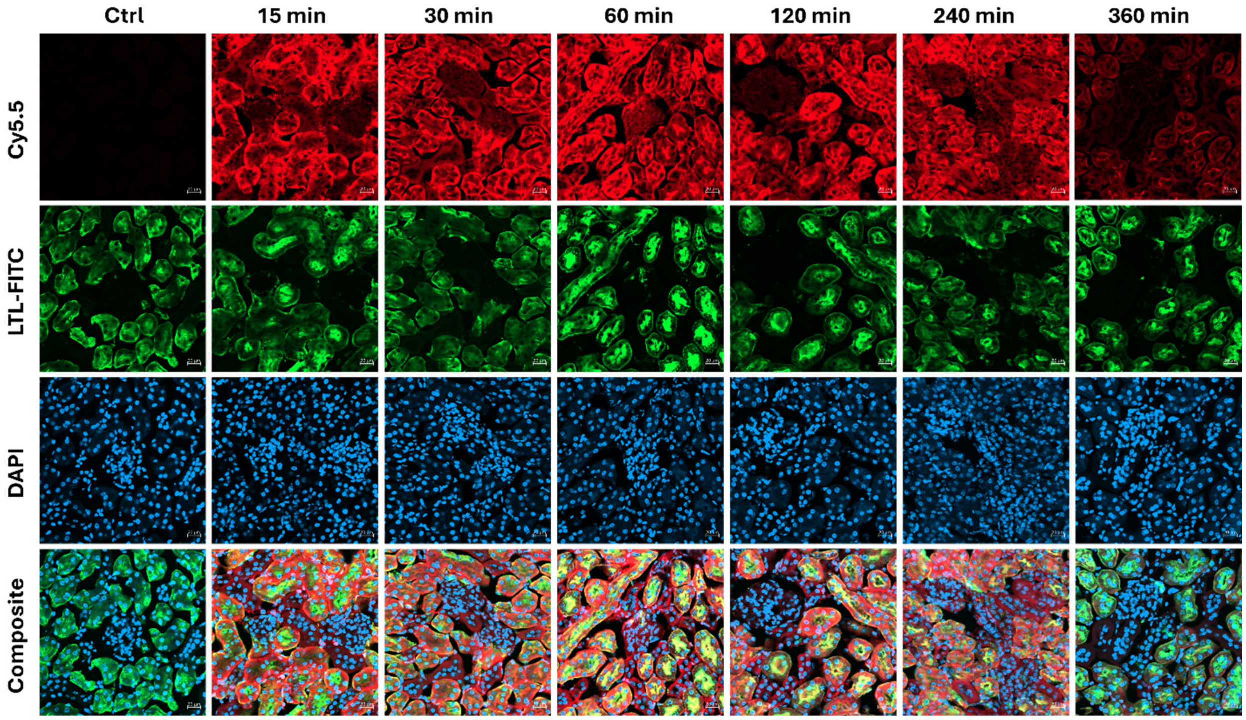

Supplemental Figure S4: Colocalization of cR11A-Cy5.5 and renal proximal tubules, visualized using Lotus Tetragonolobus lectin (LTL) staining. Kidneys were harvested from mice following an intravenous injection of cR11A (1 mg/kg) at various time points post-injection: 0 min (control), 15 min, 30 min, 60 min, 120 min, 240 min, and 360 min. LTL-FITC staining specifically marks renal proximal tubules, demonstrating the colocalization of cR11A with renal proximal tubules, but not much with glomerulus or renal distal tubules. Cy5.5 (red) represents cR11A, LTL-FITC (green) marks renal proximal tubules, and DAPI (blue) stains nuclei. The scale bar represents 20 µm. N = 3.

Supplemental Figure S5.

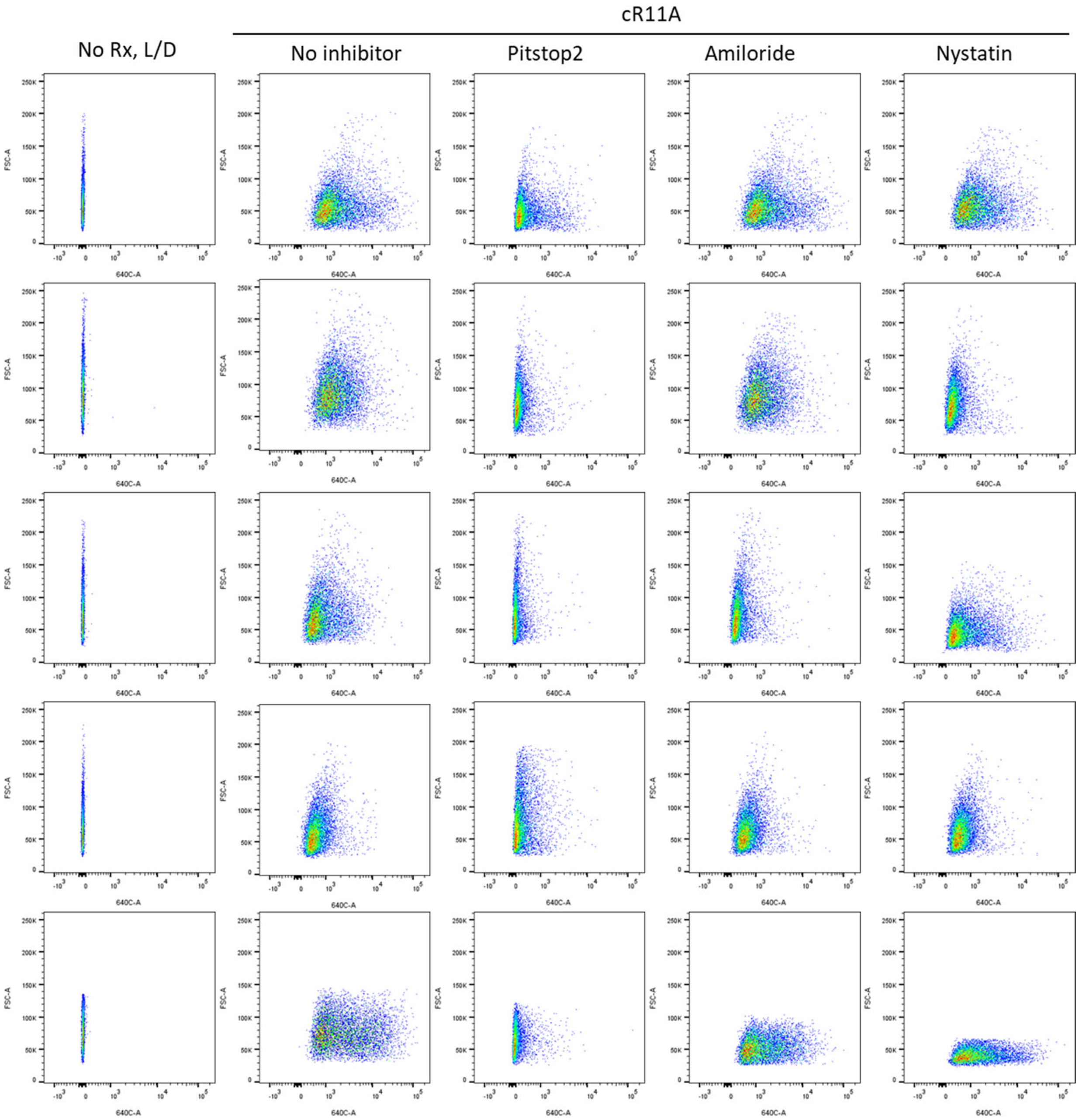

Supplemental Figure S5: Transduction mechanism study of cR11A with endocytosis inhibitors. HBE cells were starved and treated with endocytosis markers, Pitstop2, Amiloride, and Nystatin. Subsequently, cells were incubated with cR11A (1μM), with or without the inhibitors, and subjected to L/D staining. FACS data (640C-A channel) indicated the uptake of cR11A, and L/D staining assessed cell viability. Compared to group treated with cR11A alone (no inhibitor), Pitstop2 significantly reduced cR11A uptake, whereas Amiloride and Nystatin showed no notable inhibition. Results of each biological replicate (N=5) presented individually.

Supplemental Figure S6.

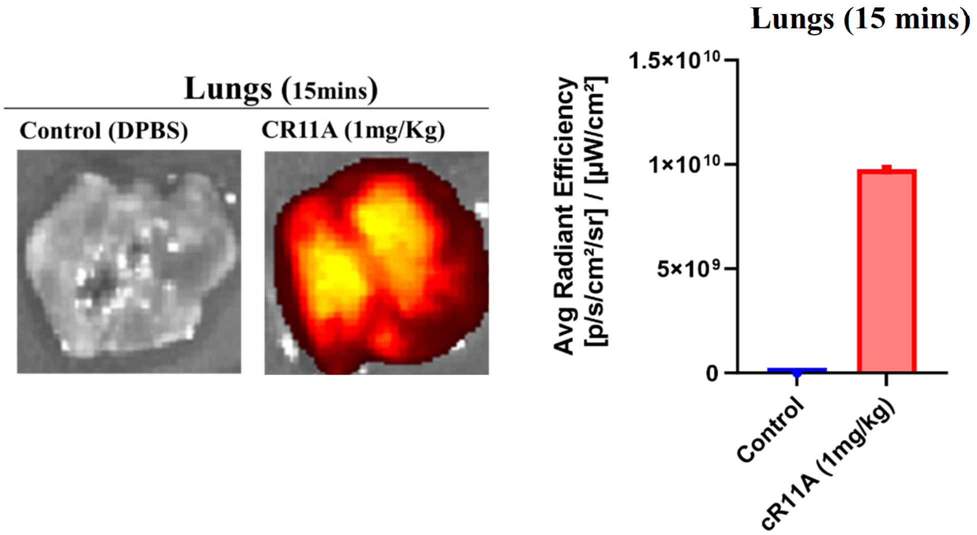

Supplemental Figure S6: Peak cR11A uptake occurs at 15 mins. Wild-type mice were injected intravenously with R11A-Cy5.5 at 1mg/Kg and euthanized, lungs harvested and IVIS imaging performed. There is robust lung uptake of the peptide at 15 mins. N=3.

Supplemental Table S1: List of antibodies for immunofluorescence staining**.**

Cell Type	Antibody Name	Vendor	Catalogue #
AT-1 cells	Monoclonal, Anti-Hop antibody (E-1)	Santacruz	sc398703
AT-2 cells	Polyclonal, Anti-ATP-Binding cassette sub- family A member 3 antibody (ABCA3)	Abcam	ab99856
Basal cells	Polyclonal, p63 antibody<br>Monoclonal Anti-Cytokeratin 5 (KRT5) antibody	GeneTex<br>Abcam	GTX102425<br>AB64081
Goblet cells	Monoclonal, Anti-Mucin 5 AC antibody(45M1)	Abcam	AB3649
Club cells	Monoclonal, Anti-Uteroglobin antibody	Abcam	AB213203,
Ciliated cells	Monoclonal, Anti-Tubulin, Acetylated antibody produced in mouse	Millipore Sigma	T7451
Ionocytes	Monoclonal, Anti-FOXI1 antibody	NOVUS	NBP2-70747AF647
Endothelial cells	Monoclonal, Anti-PECAM1 (CD31) antibody	Abcam	AB182981
Smooth muscle cells	Monoclonal, Anti- α-Smooth Muscle Actin (D4K9N) antibody	CST	19245S
Renal tubules	Lotus Tetragonolobus (Asparagus Pea) Lectin (LTL), fluorescein (FITC)	Invitrogen™	L32480

Supplemental Figure S7.

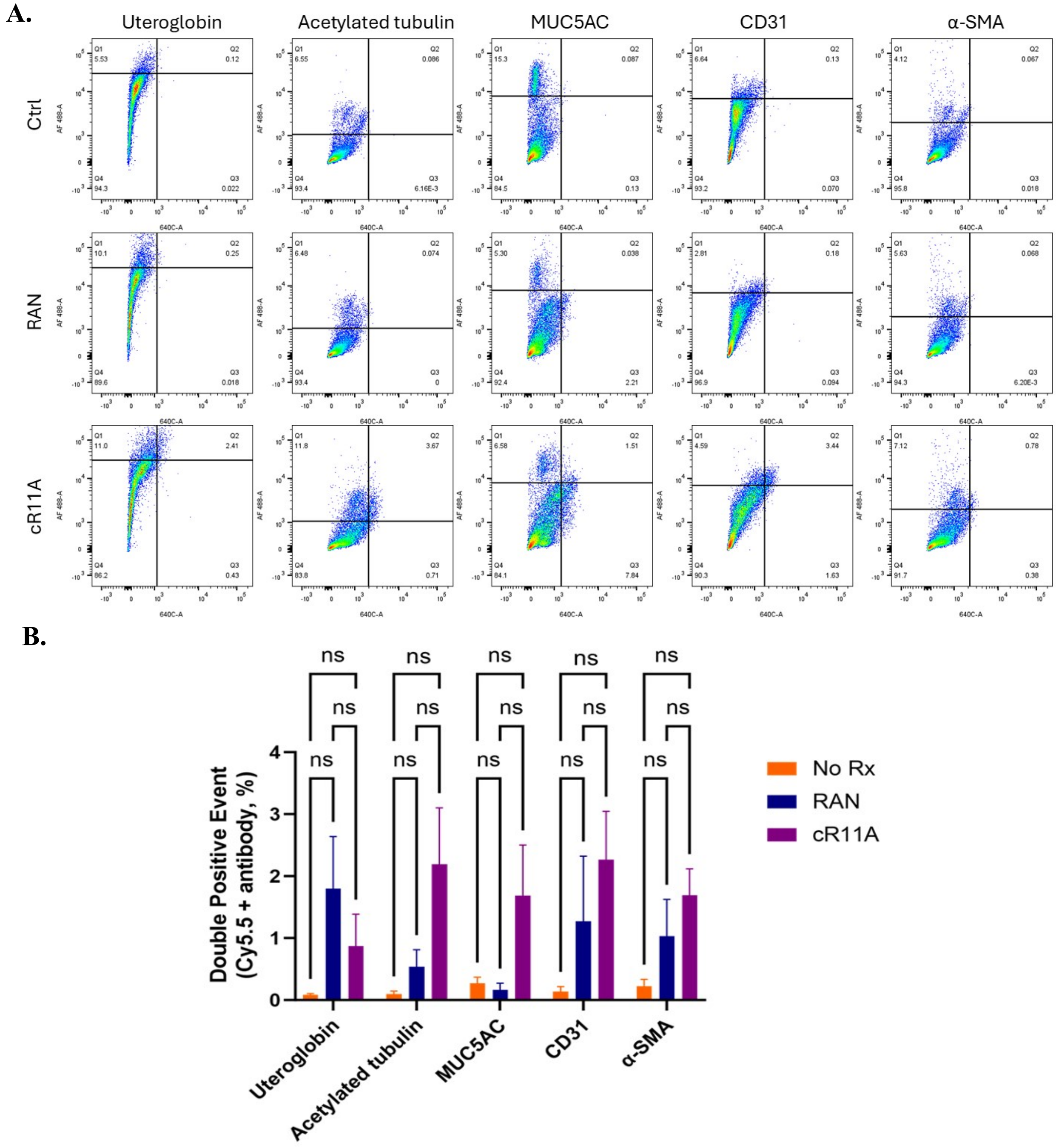

Supplemental Figure S7: Characterization of lung single cells isolated from cR11A-treated mice. Single cells from lungs of mice injected with cR11A (1mg/Kg) were isolated and stained with markers for various cell types. A) Representative FACS panels display the expression of cell-specific markers, including club cells (Uteroglobin), ciliated cells (acetylated tubulin), goblet cells (MUC5A), pulmonary endothelial cells (CD31), and airway smooth muscle cells (α-SMA). B) Statistical analysis revealed no significant differences in uptake of random peptide from cR11A. Data are presented as mean ± SEM, N = 4.

Supplemental Figure S8.

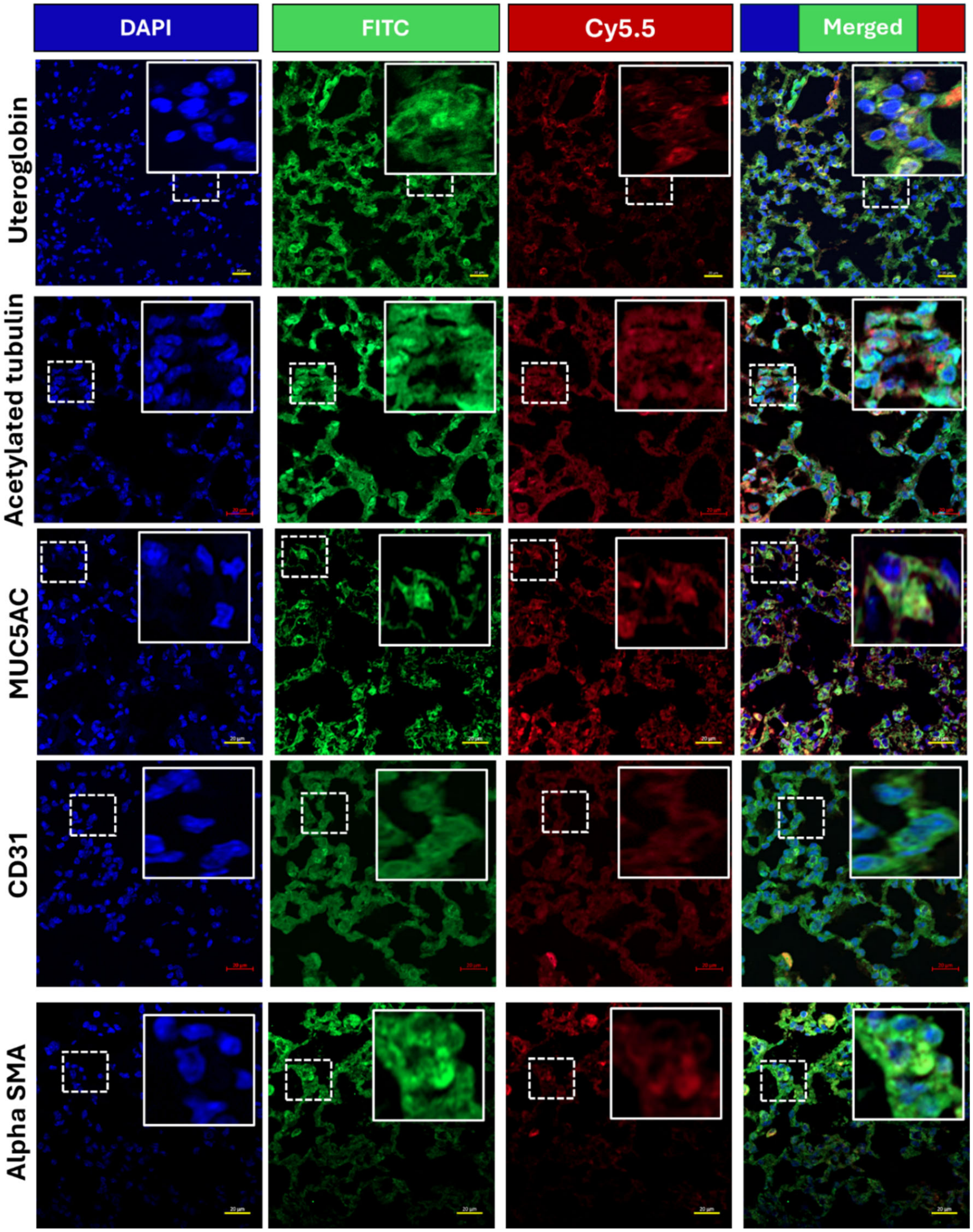

Supplemental Figure S8: Confocal micrograph of lungs from wild-type mice injected with cR11A-Cy5.5 and euthanized at 15 minutes. Minimal uptake of cR11A (red) is observed in club, ciliated, goblet, endothelial, and smooth muscle cells, as shown by lack of significant co-localization with acetylated tubulin, MUC5AC, CD31, and alpha SMA (green). Nuclei are stained with DAPI (blue). Scale bar represents 20 µm. N = 3.

Supplemental Figure S9.

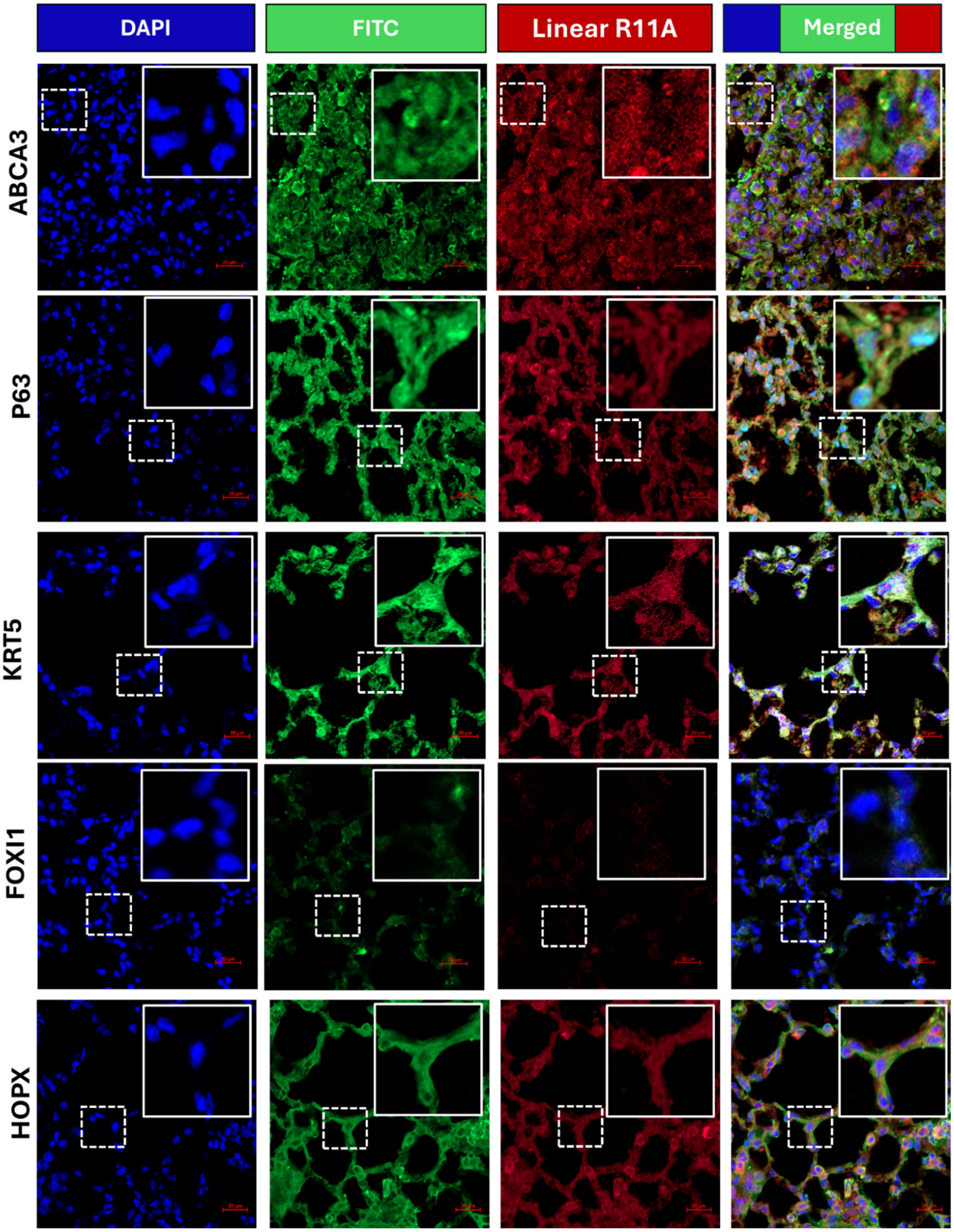

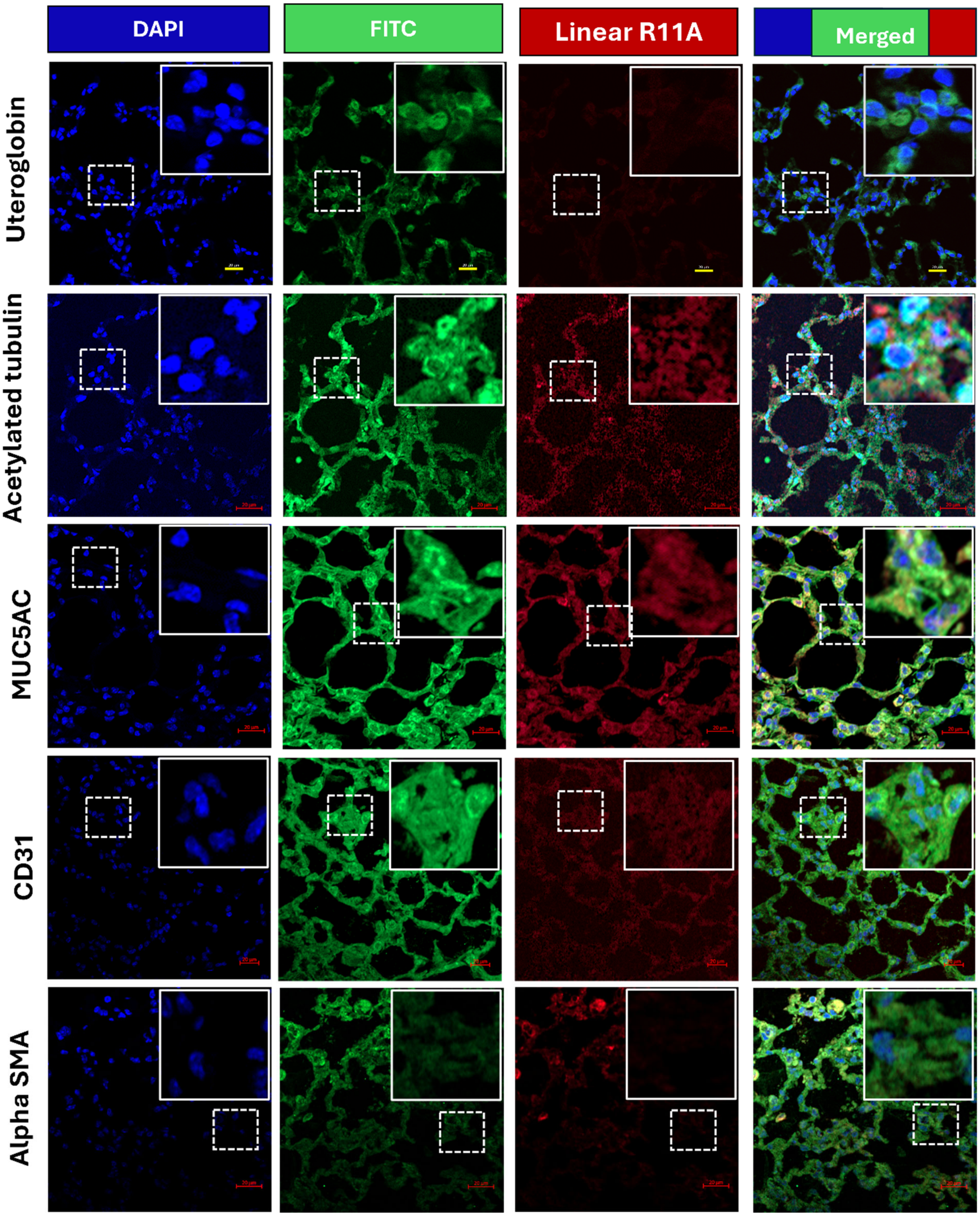

Supplemental Figure S9: Confocal micrograph of lungs from wild-type mice injected with linear R11A (10mg/Kg) and euthanized at 15 minutes. There is uptake of linear R11A (red) in same lung cell types as cR11A but in much lesser amounts, observed in AT2, p63+ cells (green), with no uptake observed in ionocytes, AT-1, club, ciliated, goblet, endothelial, and smooth muscle cells. Nuclei are stained with DAPI (blue). Scale bar represents 20 µm. N = 3.

Supplemental Table S2: List of duplex siRNA and their targets tested in our study.

Target Protein	Target Position	Target Sequence	RNA oligo, Guide	Passenger	Seed-duplex stability (Tm), guide	Passenger 2	MW-Guide	MW-Passenger
**Envelop-E1**	**70-92**	**GTGGTATTCTTGCTAGTTACACT**	**UGUAACUAGCAAGAAUACCAC**	**GGUAUUCUUGCUAGUUACACU**	**14.3**	**14.5**	**6908**	**6833**
**Envelop-E2**	**149-171**	**GTCTTGTAAAACCTTCTTTTTAC**	**AAAAAGAAGGUUUUACAAGAC**	**CUUGUAAAACCUUCUUUUUAC**	**5.5**	**7.2**	**6996**	**6738**
**Nucleocapsid-N1**	**789-811**	**TGCCACTAAAGCATACAATGTAA**	**ACAUUGUAUGCUUUAGUGGCA**	**CCACUAAAGCAUACAAUGUAA**	**13.5**	**11.8**	**6896**	**6892**
**Nucleocapsid-N2**	**1101-1123**	**GCCTAAAAAGGACAAAAAGAAGA**	**UUCUUUUUGUCCUUUUUAGGC**	**CUAAAAAGGACAAAAAGAAGA**	**5.5**	**-3.8**	**6725**	**7065**
**Spike-S1**	**977-999**	**TTGTTAGATTCCTAATATTACA**	**UAAUAUUAGGAAAUCUAACAA**	**GUUAGAUUUCCUAAUAUUACA**	**-8**	**6.9**	**6918**	**6825**
**Spike-S2**	**2260-2282**	**TTGCAATATGGCAGTTTTTGTAC**	**ACAAAAACUGCCAUAUUGCAA**	**GCAAUAUAUGGCAGUUUUUGUAC**	**5.6**	**5.6**	**6892**	**6896**

Supplemental Figure S10.

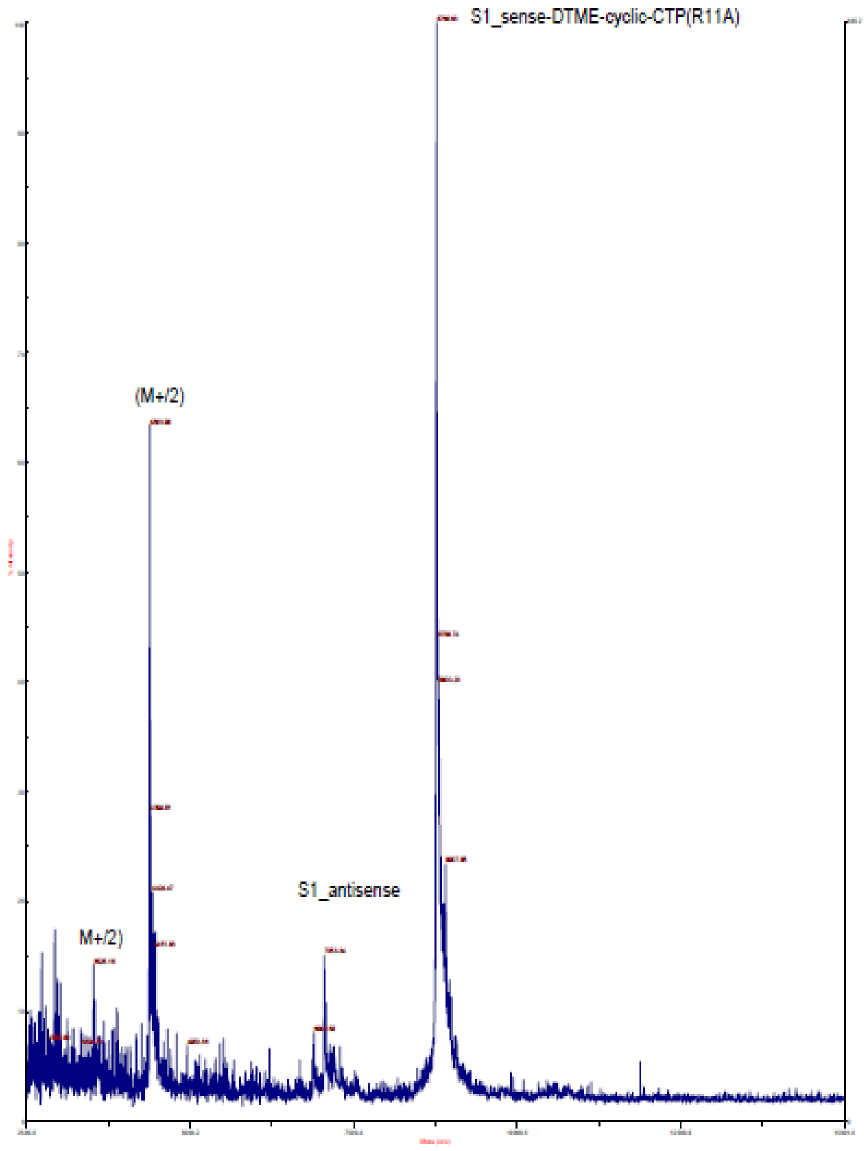

Supplemental Figure S10: MALDI-TOF analysis of cyclic R11A-siRNA-S1 showing the size and peaks of the conjugate. Sense strand-DTME-cR11A is seen as a single lateappearing peak with anti-sense strand breaking off from the conjugate due to ionization processing for MALDI. Excess peptide is seen as the earliest peak.

Supplemental Table S3: Results of VERO Cells incubated with cyclic R11A-siRNA conjugates followed by infection with SARS-CoV-2 virus.

Table S3a. Percent toxicity of cyclic R11A-siRNA conjugates on Vero 76 cells

	**Percent Toxicity**				
Conc. (μM)	**cR11A-S1**	**cR11A-S2**	**cR11A-E2**	**cR11A-N1**	**cR11A-N2**
100	0.0%	14.4%	9.7%	0.0%	4.5%
10	10.1%	2.8%	17.9%	0.0%	3.0%
1	18.1%	2.8%	17.9%	0.0%	5.2%
0.1	14.6%	2.8%	17.9%	0.0%	0.0%

Table S3b. Percent cytopathic effect of University of Pittsburgh compounds against SARS-CoV-2

	**Percent CPE**				
Conc. (μM)	**cR11A-S1**	**cR11A-S2**	**cR11A-E2**	**cR11A-N1**	**cR11A-N2**
100	57.9%	64.6%	53.7%	78.6%	60.2%
10	87.3%	89.3%	89.6%	96.9%	90.8%
1	90.1%	93.1%	95.4%	97.6%	96.8%
0.1	90.1%	100.0%	100.0%	95.5%	99.3%

CPE - Cytopathic effect

Compounds pretreated on cells for 24 hours prior to infection with SARS-CoV-2 virus.

Supplemental References

Reed, L.J., Muench, H., 1938. A simple method of estimating fifty percent endpoints. The American Journal of Hygiene 27, 493–497.
